# Arctic and Boreal Wildfires Impact Climate by Releasing Ancient Carbon and Light-Absorbing Particles

**DOI:** 10.1021/acs.est.5c17130

**Published:** 2026-04-09

**Authors:** Meri. M. Ruppel, Markus Somero, Olli Sippula, Mika Ihalainen, Juho Louhisalmi, Jarkko Tissari, Negar Haghipour, Johan Ström, Minna Väliranta, Kajar Köster, Ville Vakkari, Kerneels Jaars, Lin Huang, Rienk H. Smittenberg

**Affiliations:** † Atmospheric Composition Unit, Finnish Meteorological Institute, Helsinki 00560, Finland; ‡ Environmental Change Research Unit (ECRU), Department of Environmental Sciences, University of Helsinki, Helsinki FI-00014, Finland; § Department of Environmental and Biological Sciences, 4344 University of Eastern Finland, Kuopio FI-70211, Finland; ∥ Department of Chemistry and Sustainable Technology, University of Eastern Finland, Joensuu FI-80101, Finland; ⊥ Department of Earth Sciences, Geological Institute, ETH Zurich, Zurich 8092, Switzerland; # Laboratory for Ion Beam Physics, ETH Zurich, Zurich 8093, Switzerland; ∇ Department of Environmental Science (ACES), Stockholm University, Stockholm 11418, Sweden; ○ Atmospheric Chemistry Research Group, Chemical Resource Beneficiation, 26697 North-West University, Potchefstroom 2520, South Africa; ◆ Climate Research Division, Atmospheric Science & Technology Directorate, Environment and Climate Change Canada, Toronto, ON M3H 5T4, Canada; ¶ Swiss Federal Institute for Forest, Snow and Landscape Research WSL, Zuercherstr. 111, Birmensdorf 8903, Switzerland

**Keywords:** high-latitude fires, peat, combustion experiment, carbonaceous particles, carbon isotopes, carbon emissions, light-absorbing particles, wildfire climate impacts

## Abstract

Climate warming induced wildfires are rapidly increasing at high latitudes, yet their climate impacts remain poorly understood. These deeply smoldering fires may release long-stored carbon and thus perturbate the global carbon cycle and further emit light-absorbing carbonaceous particles enhancing snow and ice melt after deposition. We newly investigate the carbon isotopic and light-absorbing characteristics of carbonaceous particles produced in laboratory combustion experiments on Arctic-boreal peats and compare these with biomass from boreal forest and savanna environments. We provide the first observational evidence that boreal and especially Arctic peat smoldering may release millennial-aged carbon into the atmosphere, which upsets radiocarbon-based source attribution, separating fossil-fuel-derived sources from modern biomass. Moreover, above- and below-ground material combust differently, and hence the fraction of modern carbon (F^14^C), i.e., the average age, of the original biomass and the produced carbonaceous particles may differ. Furthermore, we show that peat smoldering produces significant amounts of Brown Carbon, which absorbs light at a similar magnitude to Black Carbon in these samples. Our results indicate that the increasing number of Arctic-boreal peat fires may exacerbate Arctic warming more than previously estimated.

## Graphical abstract

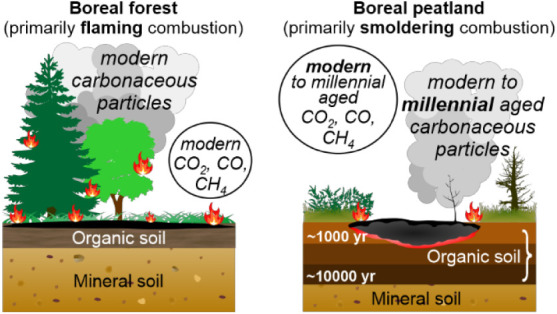

## 1 Introduction

Peatlands store approximately 30% of the global total soil carbon,^1^ with 80% of this carbon contained in Arctic peatlands.^2^ An emerging concern caused by accelerated climate change and permafrost thaw is the rapid increase in Arctic peatland fires,^2–7^ which have already expanded to the Siberian Arctic Ocean coast,^8^ Greenland,^9^ and Alaskan tundra peatlands.^10,11^ These fires occur along with record-breaking boreal forest fires raging in Canada.^12^ Peat fires may smolder for weeks and months, releasing massive amounts of, potentially ancient, carbon,^3,4,6^ which may transform them from a major carbon sink into a net carbon source into the atmosphere.^7,13^ While forest fires predominantly consume surface vegetation, smoldering peat fires propagate vertically into the soil organic matter, leading to the largest fires on Earth in terms of mass of fuel consumed per unit surface.^13–17^ The fast spread of high-latitude fires has led to urgent calls to better understand their drivers and environmental consequences.^4–6^

In addition to globally significant greenhouse gas emissions,^7,18–20^ peatland fires release abundant particulate matter, but relatively few investigations have quantified the latter.^21–25^ Smoldering peat fires may emit six times more aerosol mass per unit carbon combusted compared to flaming (grassland and forest) fires.^7,26^ Wildfires are a major source of Black Carbon (BC), which is the strongest light-absorbing particulate with a large positive global radiative effect.^27,28^ Simultaneously, wildfires release abundant Organic Carbon (OC), which can have a cooling effect either directly by scattering solar radiation or indirectly by modulating cloud properties.^27^ However, increasing evidence of substantial light-absorbing OC, i.e., Brown Carbon (BrC), being emitted in wildfires, suggests that the atmospheric net effect of biomass burning plumes is warming,^29–33^ as specifically shown for boreal^23^ and Indonesian^34^ peat combustion, while the properties and atmospheric lifetime of BrC vary depending on, for instance, combustion characteristics, biomass type, and moisture content.^30,35^ Moreover, BC and BrC decrease surface albedo when deposited on snow and ice, further accelerating Arctic climate change.^27,36,37^ Consequently, increasing high-latitude peatland fires are of great concern in the vicinity of snow- and ice-covered surfaces.

To assess the importance of peat-smoldering-derived particles for climate change, it is essential to distinguish these particles from other emission sources. Dual-carbon isotope (radiocarbon (F^14^C) and stable carbon (δ^13^C)) analyses have allowed estimation of the relative contributions of BC and OC emission sources in atmospheric and snow samples.^38–40^ The radiocarbon content reveals whether the particles originate from modern biomass or fossil fuel combustion,^41^ while the δ^13^C signature allows further classification of sources to biomass, coal, oil, or natural gas.^42^ These detailed source constraints facilitate observation-based evaluation of bottom-up BC emission inventories used in climate modeling.^40^ However, dual-carbon isotope source attribution cannot distinguish peat-fire-derived particles because their stable carbon isotopic endmember has not been determined, but it most likely is similar to the endmember of carbonaceous particles from biomass combustion in general.^42–44^ Additionally, the radiocarbon content of peat-derived BC and OC depends on the age of the combusted peat. Thus, in regions where peat combustion is a significant source of carbonaceous particles, such as Indonesia^45–48^ and increasingly the Arctic and boreal region,^3,4,6^ the dual-carbon isotope source attribution may overestimate fossil (oil, coal, and natural gas) contributions to BC and OC. This may lead to inadequate political decision-making regarding the most important BC emission sources.^49,50^

In this study, we use peat collected from eight boreal and Arctic Eurasian peatlands (Figure 1) to characterize the dual-carbon isotope (F^14^C and δ^13^C) signature of the peat and the carbonaceous particles released from its combustion under flaming and smoldering conditions. We compare our isotope results from peat with similar data gained from boreal forest surface and savanna vegetation (Figure 1). We show the first findings on the age of particulate carbon released during combustion experiments that simulate natural peat burning conditions. We indicate the pronounced and possibly previously underestimated climate impact of Arctic and boreal peat wildfires, specifically by their release of ancient carbon and light-absorbing particles into the atmosphere. Furthermore, our findings underscore the difficulty of distinguishing peat-fire-derived carbonaceous particles from other biomass combustion sources based on the δ^13^C endmember, implying that alternative analytical methods, such as organic compound fingerprints, are required for reliable source apportionment and climate impact assessment of biomass combustion-derived particles.

**Figure 1:**
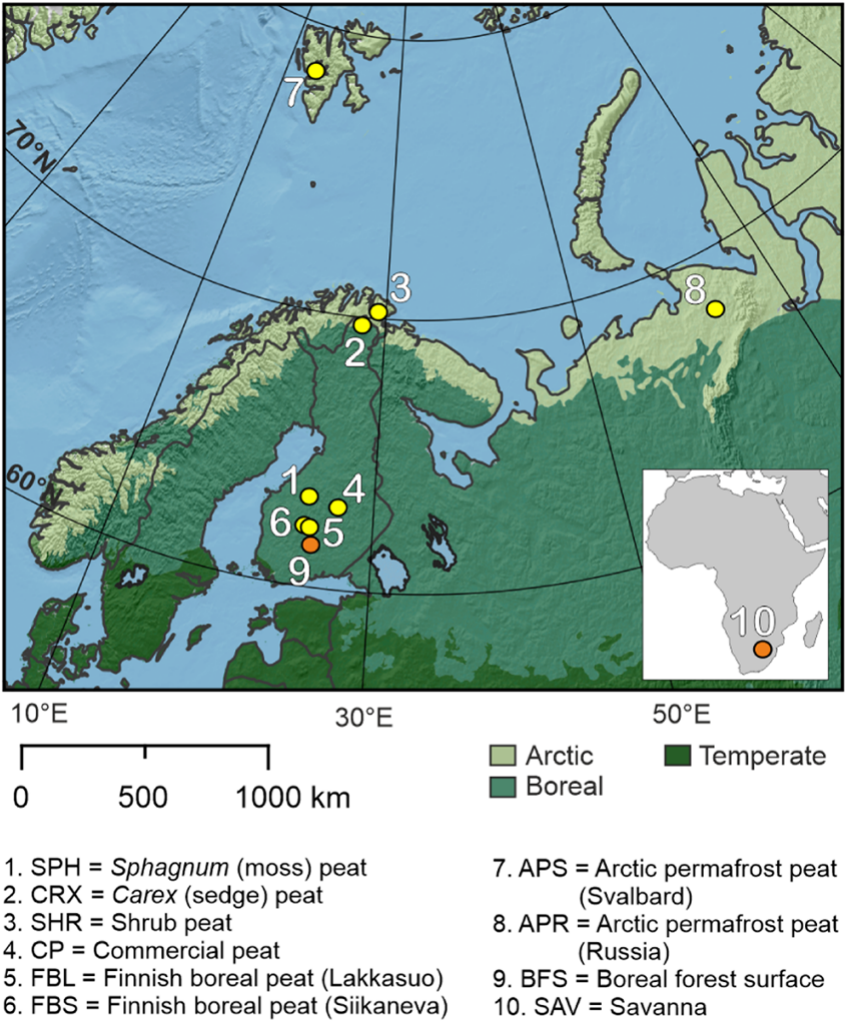
Location of the collected biomass samples. The numbering relates to the numbers given in Table S1. Yellow dots represent peat collection sites, while orange dots represent surface biomass sampling sites. The Arctic, boreal, and temperate biomes are presented in different colors.

## 2 Methods

### 2.1 Biomass Sample Collection and Acquisition

In a data set of over 1000 measurements from North American wildfires (boreal forest, tundra, and peatland) between 1983 and 2016, the recorded burn depth ranges between 0 and 35 cm, with a rough average of 15 cm,^51^ and burn depths generally increasing to the north.^52^ In natural wildfires, high-temperature flaming and low-temperature smoldering occur in tandem, as above surface material mainly combusts by flaming and below-surface material by smoldering.^14,15,25^ Thus, to separately assess the particles formed in flaming and smoldering combustion, biomasses were collected with different strategies. Surface samples of 10–20 kg were collected in summer 2021 with a shovel from the top about 15–20 cm of peat (susceptible to flaming combustion) from three boreal to subarctic permafrost peatlands in northern Fennoscandia (Samples 1–3, Figure 1, Table S1) and were packed into plastic bags. To study the influence of vegetation composition and potentially varying decomposition degree and age of the organic material on the particles released during combustion, three different peatland sites were chosen: 1. *Sphagnum*-moss peat (SPH) from northern Finland, 2. *Carex*-peat (CRX) from northern Finland, and 3. Shrub-peat (SHR), which contains roots and other woody material with some *Sphagnum*-peat from northern Norway (Figure 1, Table S1).

Second, deeper peat profiles (30–50 cm) collected in 2018 for other studies were available from two Finnish boreal peatlands (Lakkasuo (FBL) and Siikaneva (FBS)), a Svalbard Arctic permafrost peat bank (Alkhornet, APS), and a northern Russian permafrost peatland (Rogovaya, Komi Republic, APR) (Figure 1, Table S1). The profiles were collected with a box soil corer and stored frozen in plastic wrapping and represent soil layers susceptible to smoldering combustion.

To compare the carbon isotopic composition and light-absorption characteristics of natural peat (collected from the fire-susceptible layers) with fuel peat (collected from an unknown peat deposit depth), commercial peat briquettes originating from boreal central Finland were purchased (CPFa+b and CPS, Figure 1, Table S1). Moreover, to compare the results gained from peat samples with other vegetation types, previously collected Finnish boreal forest surface (samples from Scots pine forest, including vegetation, litter, and soil organic layer, BFS) and South African savanna (SAV) samples were used for this study (Figure 1, Table S1).

### 2.2 Combustion of Biomass and Production of Particulate Matter Filter Samples

The biomasses were dried (or received in a dry state), ensuring a similar moisture content for all samples, with no further pretreatment before the combustion experiments. The moisture content and elemental composition of the samples were determined by methods and results outlined in Supporting Information S1 and Table S2. To gather information on the carbonaceous particles released over the full combustion cycles under smoldering and flaming conditions, the samples were combusted by two different combustion setups to produce Particulate Matter (PM) collected on filters at the University of Eastern Finland (UEF), Fine Particle and Aerosol Technology Laboratory. In the first combustion experiment, the surface peat samples SPH, CRX, and SHR and commercial peat (CPFa+b) were combusted in a closed combustion chamber with open air intake to reach relatively high combustion temperature and *primarily* flaming combustion, with less prevalent smoldering phases. This closed experimental setup simulated flaming combustion common for surface vegetation^25^ that can reach high temperatures and is referred to as the “flaming” setup from here on. In contrast, the second experiment on the peat profile samples FBL, FBS, APS, and APR, and additional biomasses CPS, BFS, and SAV, represented open biomass burning at low temperatures, which therefore resulted in *primarily* smoldering conditions. This “smoldering” experiment included only short flaming phases and simulated natural northern wildfires that can reach deeper soil layers,^14,25^ such as those present in profile samples FBL, FBS, APS, and APR. Details on the experimental setups are given in S2, and details for the calculation of the combustion efficiency and particle emission factors are provided in S3.

### 2.3 Total, Organic, and Elemental Carbon Quantification from Filters

The concentrations of the biomass combustion-derived carbonaceous particles on the PM filters were determined using a thermal-optical carbon analyzer (Sunset Instrument, Model 5L)^53^. In this conventional thermal-optical method, the carbonaceous aerosol fractions collected on filters are separately quantified based on their temperature-specific volatilization, controlled temperature and redox conditions, and optical correction for pyrolytically generated carbon (charring).^53^ Here, we used the ECT9-protocol, recently developed for the collection of OC and Elemental Carbon (EC, thermal-optical proxy for BC) fractions, to determine the carbon isotopic composition of filter samples,^54^ with further details given in S4.

### 2.4 Brown Carbon (BrC) Analysis from Filters

Light-absorbing organic carbon, i.e., Brown Carbon (BrC), was estimated on the filters with a DRI 2015 Series 2 Multi-Wavelength Thermal/Optical Carbon Analyzer (Aerosol Magee Scientific), which monitors the optical transmittance and reflectance of the sample simultaneously at 7 wavelengths, uniquely allowing BrC quantification from the OC fraction.^55^ BrC cannot be directly quantified as mass with the DRI instrument, but the absorption on the filter between EC and BrC can be expressed as percentages, as further explained in S5.

### 2.5 Preparation for Radiocarbon and Stable Carbon Isotope Analysis

The OC and potentially pyrolyzed OC were removed from the filters by running a truncated version of the ECT9 temperature protocol on the Sunset Instrument, stopping after the first two steps in the helium phase.^54^ This resulted in filters containing only EC, intended for radiocarbon and stable carbon isotope measurements.

### 2.6 Radiocarbon and Stable Carbon Isotope Determination of Total and Elemental Carbon (TC and EC)

The radiocarbon (^14^C) and stable carbon isotope (^13^C) content of the original biomass material, the combustion-derived particulate total carbon (TC) filter, and the filter samples containing only the isolated EC fraction were separately analyzed with an integrated, online EA-IRMS/AMS system in routine use at ETH Zürich.^56^ The system comprises an elemental analyzer (EA, Elementar) and a stable isotope ratio mass spectrometer (IRMS, Isoprime) connected to a gas interface system (GIS, Ionplus) and a Mini Carbon Dating System (MICADAS, Ionplus^57,58^ ) analyzing the ^14^C content with accelerator mass spectrometry. When the carbon content of the samples was below 20 μg, all volatilizing carbon was used for ^14^C analysis, and the ^13^C contents were determined separately by IRMS if any material was available.

Radiocarbon content is measured as ^14^C/^12^C ratios and reported as Fraction Modern (F^14^C), as described by Reimer et al.^59^ The measured ^13^C/^12^C ratios are reported in δ^13^C notation relative to the Vienna Pee Dee Belemnite (VPDB) standard.

Several blank filters were prepared for all the different types of filters used in the biomass combustion experiments, and their F^14^C and δ^13^C values were determined as described above. The blank values were subtracted from the respective isotope measurement results. Correction for stable carbon isotopic fractionation is outlined in S6.

## 3 Results and Discussion

### 3.1 Emission Factors of Total, Organic, and Elemental Carbon (TC, OC, and EC) and Combustion Efficiency of the Biomass Combustion Experiments

Our biomass combustion experiments highlight how variable combustion conditions affect particulate matter emissions. The TC and OC emission factors were, on average, around four times higher in the smoldering compared to the flaming-dominated combustion experiment, while the EC emission factor remained similar in both experiments (Table 1). As the emission factors vary both due to different combustion conditions and the variable inherent combustion behavior of the biomasses, the influence of burning conditions is best exemplified by the commercial peat, which produced over 6-fold OC emissions but five-times lower EC emissions during the smoldering experiment (CPS) compared to its flaming combustion (CPFa, CPFb) (Table 1). These results concur with previous findings, indicating that smoldering combustion releases several times more carbonaceous particle emissions compared to flaming combustion, mainly in the form of OC.^7,23,26,60,61^

**Table 1:** Emission Factors (g/kg) of Total, Organic, and Elemental Carbon (TC, OC, and EC, Respectively) Produced during Flaming- and Smoldering-Dominated Biomass Combustion Experiments and Measures of Combustion Efficiency^a^

		**Emission factors**				
Dominant combustion setup	Sample ID	TC (g/kg)	OC (g/kg)	EC (g/kg)	EC/TC	MCE
Flaming	**1.** *Sphagnum* peat (**SPH**)	0.29	0.2	0.09	0.31	0.97
Flaming	**2**. *Carex* peat (**CRX**)	12.66	12.38	0.28	0.02	0.94
Flaming	**3.** Shrub peat (**SHR**)	1.08	0.5	0.58	0.53	0.97
Flaming	**4a.** Commercial peat (**CPFa**)	3.98	2.58	1.4	0.35	0.96
Flaming	**4b.** Commercial peat (**CPFb**)	3.3	2.37	0.93	0.28	0.97
Smoldering	**4c.** Commercial peat (**CPS)**	16.54	16.28	0.26	0.02	0.8
Smoldering	**5.** Finnish boreal peat (Lakkasuo) (**FBL**)	16.09	15.62	0.47	0.03	0.83
Smoldering	**6.** Finnish boreal peat (Siikaneva) (**FBS**)	19.2	18.63	0.57	0.03	0.78
Smoldering	**7.** Arctic permafrost peat (Svalbard) (**APS**)	7	6.88	0.12	0.02	0.82
Smoldering	**8.** Arctic permafrost peat (Russia) **(APR**)	9.97	9.77	0.2	0.02	0.89
Smoldering	**9.** Boreal forest surface (**BFS**)	14.91	13.96	0.95	0.06	0.79
Smoldering	**10.** Savanna (**SAV**)	30.43	28.55	1.88	0.06	0.74

a The ratio of EC to TC demonstrates burning efficiency during the experiment with higher values indicating more complete combustion, while Modified Combustion Efficiency (MCE) values above 0.9 suggest flaming burning and values below 0.9 represent smoldering combustion.

The average OC emission factor obtained for our smoldering peat experiment (Samples 4c-8, average 13.77 g/kg) concurs with or is a little higher than previously reported for Alaskan and Siberian peat^23^ and peat smoldering in general.^25,62^ In our experiment, the TC emissions were higher from the smoldering savanna tree than from peat combustion (Table 1), but in nature, savanna fires are rather flaming than smoldering.^14,25,62^ Generally, our results underline the elevated particulate carbon emissions in low-temperature smoldering fires common in Arctic and, to a lesser extent, boreal environments, compared to flaming-dominated wildfires more likely to occur in temperate and savanna environments.^14,25,62^

In addition to the calculated Modified Combustion Efficiency (MCE) indicating flaming combustion with values above 0.9 for the first experiment and smoldering with values below 0.9 for the second experiment (Table 1), differences in the combustion conditions between the flaming and smoldering experiments are also evident in the EC to TC ratios (Table 1). During the flaming-dominated experiment (Samples 1–4b), relatively higher EC/TC ratios were obtained, on average 0.3 (range 0.02–0.53). The CRX sample diverges with an EC/TC ratio of 0.02 (and a comparably significantly higher OC emission factor), suggesting an inherently smoldering burning behavior of this biomass, i.e., sedge vegetation from a wet mire, in contrast to the other samples in the flaming experiment that consisted mainly of *Sphagnum* moss and shrub remains. The EC to TC ratio obtained during the smoldering experiment (Samples 4c-10) is, on average, 0.03 (range 0.02–0.06), i.e., ten times lower than in the flaming experiment. These results concur with general findings of EC being produced under flaming conditions, whereas OC is predominantly produced in smoldering combustion phases characterized by relatively higher OC to EC ratios.^23,60^

### 3.2 Radiocarbon Age of Combusted Biomass vs the Age of Total Particulate Carbon (TC) Collected on Combustion Filters

Our combustion experiment revealed notable differences between the age of the original biomass and that of the particulate carbon collected on the combustion filters. Moreover, boreal and arctic peats appear to exhibit different combustion behaviors, reflected in diverging ages of the released carbon phases (i.e., gases, OC, and EC). These observations can be explained only via understanding of the composition of peat profiles, which consist of plant remains of variable species, decomposition degree, and age with depth (see S7, and Figures S6 and S7), which may lead to variable propensity of the organic material present at each depth for smoldering and flaming combustion.

Our combusted biomass samples consisted of differently aged material, both between the samples and internally. According to the radiocarbon data, the biomass collected from the top 15–30 cm of peat from the peatlands SPH, CRX, FBL, and FBS was modern peat (F^14^C ≥ 1) (Figure 2, Table S4). Also, the savanna sample (SAV) can be considered modern, with an estimated age of 50 years (fraction of modern carbon, i.e., F^14^C = 0.99). We also expect that the Norwegian shrub peat (SHR) and boreal forest surface samples (BFS) are modern, while this cannot be confirmed, as no sample of the original biomass was left for radiocarbon dating. In contrast, the commercial peat briquettes (CP samples) originate from peat layers with an *average* ^14^C age of approximately 1500 years (F^14^C = 0.83) (Figure 2; Table S4). As hypothesized, the Arctic peat profiles (APS and APR) contained, on average, older peat material than the modern subarctic and boreal samples (SPH, CRX, FBL, and FBS), although being collected at similar depths. The *average* age of the top 30 cm of Arctic Svalbard peat (APS) was about 1400 yr (F^14^C = 0.84), which indicates that younger peat was present in the surface layers of the profile and older in the bottom parts (S7, Figures S6, S7). The 50 cm thick Arctic Russian peat core (APR) showed an average ^14^C age of about 2400 yr (F^14^C = 0.74) but represents a considerably deeper profile than the others (Figure 2; Table S4).

**Figure 2:**
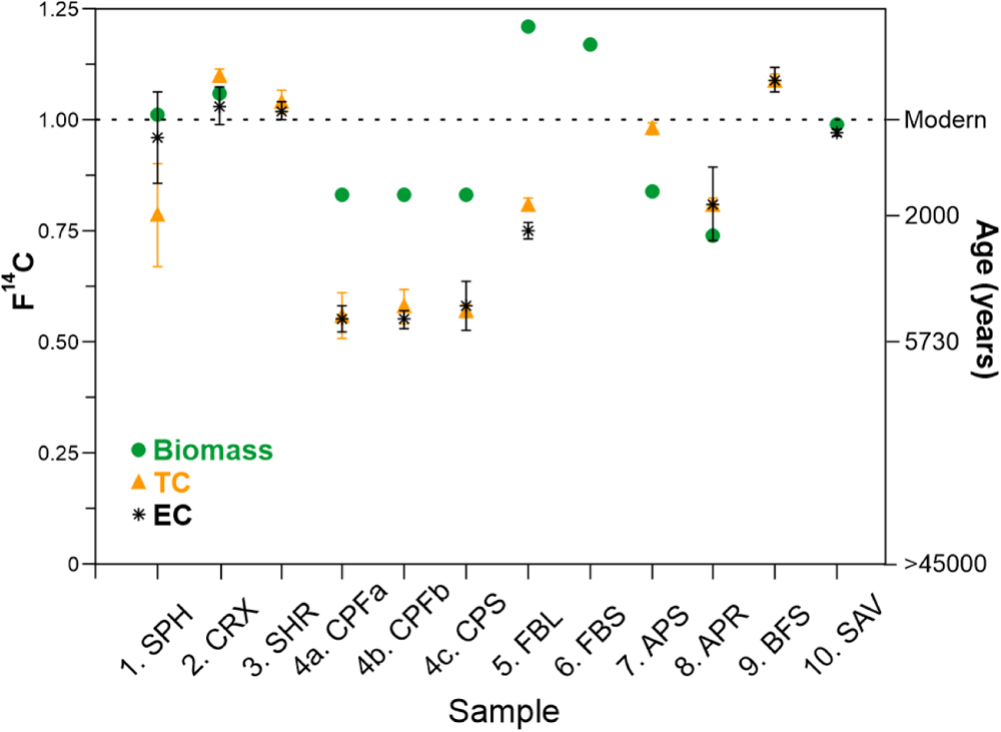
Radiocarbon contents of original biomass, and particulate Total Carbon (TC) and Elemental Carbon (EC) fractions on filters from the combustion experiments, expressed as Fraction Modern (F^14^C) (left axis) and radiocarbon age (right axis). Error bars express 2s analytical uncertainties. Radiocarbon data for the original biomass are missing for SHR and BFS, for the TC of FBS, and for the EC of FBS and APS due to a lack of sufficient sample material.

Comparison of the radiocarbon ages of the particulate TC collected on filters during the combustion experiments with the age of the original biomass highlights key findings of our study. The age of TC is either similar to or deviates significantly from the age of the original biomass, being either younger or older (Figure 2, Table S4). For the six samples where the comparison can be made, one sample (CRX) shows similar ages of the original biomass and TC collected on the filters, whereas the remaining samples (SPH, CP, FBL, APS, and APR) show remarkable differences. For some boreal peats (SPH, CPFa, CPFb, CPS, and FBL), the TC is significantly older than the original biomass, whereas in the case of Arctic peats (APS, APR), the TC is younger than the original biomass.

The different relations in ages derive from the heterogenic biogeophysical composition of the original biomass. The samples’ vertical layers contain plant remains of increasing age and degree of decomposition with depth (see S7) and are generally more prone to smoldering with increasing depth due to inherent variations in the ignition temperature of the materials present at different depths.^14^ This potential heterogeneity within each sample material and its combustion behavior led to variable ^14^C ages and variable amounts of OC and EC captured on the filters across smoldering and flaming combustion phases and the observed differences in radiocarbon age between the original biomass and the particles captured from its combustion.

Previous biomass combustion experiments have shown that gas- and particulate phase emissions are produced in different proportions in flaming vs smoldering conditions. Flaming combustion dominates total carbon emissions, releasing mainly carbon dioxide (CO_2_), and relatively minor particulate matter emissions.^43,60^ In contrast, smoldering combustion yields higher levels of carbon monoxide (CO) and other incompletely oxidized pyrolysis products, such as PM, of which emission factors are severalfold in smoldering combustion compared to flaming.^14,43,60,61^ Generally, peat combustion produces manifold PM emissions per unit mass compared to other biomass since peat dominantly smolders, while other biomass primarily combusts through flaming.^7,14,22,25,61,62^ The different abundance of combustion products in the gas and particle phases during the combustion of biofuels concurs with our observations for our smoldering combustion experiment discussed by Schneider et al.^63^ In our experiment, smoldering-dominated combustion resulted, on average, in approximately four times higher carbonaceous particle emissions than flaming-dominated combustion (Table 1).

Furthermore, in natural wildfires, flaming combustion primarily consumes young surface vegetation, while smoldering consumes older soil organic matter.^25^ Young and living biomass, such as leaves, tree parts, and other above-ground material, has a low density and burns fast in wildfires at higher temperatures^64,65^ than oxygen-depleted, pyrolytic smoldering of below-surface organic soils, stumps, and roots.^66^ The flaming combustion of surface biomass is explained by its higher O:C and H:C ratios compared to peat, resulting in a higher volatile fraction of the fuel during its combustion.^65^ Differences in boreal fire dynamics have been reported even on a continental scale, with dominant tree species favoring high-intensity flaming crown fires in North America and lower-intensity, more commonly smoldering, surface fires in Eurasia.^67^

Each of our combustion experiments included both flaming and smoldering combustion. Visual observation indicated that above-ground young material, such as twigs, shrubs, cones, and leaves, was quickly consumed by flames at the beginning of the combustion, efficiently yielding CO_2_ but little PM. In contrast, partially decomposed older below-ground material was consumed more slowly under smoldering conditions prevalent in the latter stage of each experiment, producing high amounts of PM efficiently collected on the filters.

Consequently, the observed older ages of TC compared to the original biomass for SPH, CPFa, CPFb, CPS, and FBL (Figure 2, Table S4) are explained by a combination of these two processes: particulate carbon emissions are formed primarily by smoldering, and smoldering preferentially consumes deeper, i.e., older, soil layers. In contrast, flaming combustion primarily consumes modern vegetation while generating relatively insignificant particulate carbon amounts, and carbon is emitted predominantly as gases. Interestingly, comparison of flaming vs smoldering experiments on the commercial peat (CPFa and CPFb with CPS; Figure 2, Table S4) shows that, irrespective of the dominant combustion phase, the above-described age separation between original biomass and produced TC occurred during both experiments, although with different emission factors (Table 1). On the other hand, for CRX, SHR, and BFS, the age of particulate TC collected on the filters is modern, as is the original dated biomass sample (CRX) or is assumed to be (SHR and BFS, with no dating available, Figure 2; Table S4). We expect that these surface biomass samples were originally most homogeneous in age (modern) of all studied biomasses and did not include older peat material.

In contrast, the above-described relationship of smoldering producing particulate carbon emissions from older-aged materials and flaming yielding mainly gas products from younger-aged biomass does not hold for the Arctic peat samples APS and APR. For these, an opposite relation is observed, as the older Arctic biomass in our mixed profile samples appears to be more prone to flaming compared to younger material, which is in contrast to the general assumption of surface material predominantly flaming and below-ground material smoldering.^14,17,25,69^ This reversed relation is attributed to differences in the organic geochemical composition between the Arctic (APS, APR) and boreal (FBL, FBS, and BFS) samples. Lower temperatures and therefore lower microbial activity in the Arctic permafrost peats cause their slower decomposition.^70^ Due to the lower degree of decomposition, the Arctic permafrost peats contain a higher abundance of aliphatic compounds^70^ that possess a lower ignition temperature than aromatic structures more common in boreal peats.^63^ The Svalbard peat (APS) stems from the coldest sampled region, with consequently the slowest biological decomposition of the peat.^70^ Our combustion experiment results of a significantly younger age of TC collected on the filters (∼170 years, F^14^C = 0.98) compared to the original biomass (∼1400 years, F^14^C = 0.84) of APS (Figure 2, Table S4) indicate that younger biomass present in this peat profile sample smoldered most efficiently, while the older material combusted flamingly, resulting in a much lower amount of old carbon captured as particulate matter. The same conclusion can be drawn from the Russian Arctic peat sample (APR; biomass: ∼2400 years, F^14^C = 0.74; TC: ∼1700 years, F^14^C = 0.81; Figure 2, Table S4) which is from a permafrost peatland from a somewhat warmer climate with higher microbial activity than the Svalbard peat. The flaming combustion of older Arctic peat material rich in aliphatic compounds is in line with our observations of small flames during the combustion of the Arctic samples (APS, APR), contrary to the boreal peat samples (FBL, FBS) combusted under the same conditions.^63^ Consequently, it seems that due to the low decomposition of the Arctic peat, it ignites at lower temperatures and burns more easily with a small flame (producing relatively high CO_2_ emissions^63^) than the boreal samples, and therefore, also the older carbon is lost primarily as gases from the Arctic samples. In natural Arctic wildfires, flaming combustion of deeper soil layers could potentially occur after the fire has combusted off the surface material, such as the top 20 cm of organic material in Alaskan tundra fires,^10,11^ and thereby deeper and older layers become exposed to oxygen.

Altogether, our results indicate that plant remains of different decomposition degrees are inherently variably prone to flaming and smoldering in a biomass sample containing material of a wide age range. This leads to various fractions of gas and particulate carbon emissions and their respective ages from such material (Figure 3). It appears that in relatively well-decomposed boreal peat, the youngest biomass combusts by flaming, emitting carbon mainly as gases. In contrast, the older and thus more decomposed boreal material slowly smolders, emitting comparably old carbon dominantly as particulate carbon effectively captured on filters. Arctic peats, on the other hand, contain less-decomposed biomass with a high abundance of aliphatic compounds, resulting in lower ignition temperatures (Figure 3). Consequently, in Arctic peat, the relationship between the carbon phase (gases vs particulate) released in flaming versus smoldering combustion and its age is not as straightforward as in boreal peats. However, in total, the carbon emitted as both gases and particles from arctic peat fires is older than from boreal fires, as older biomass is present at the same fire-susceptible depths in Arctic than in boreal environments (Figure 3).

**Figure 3:**
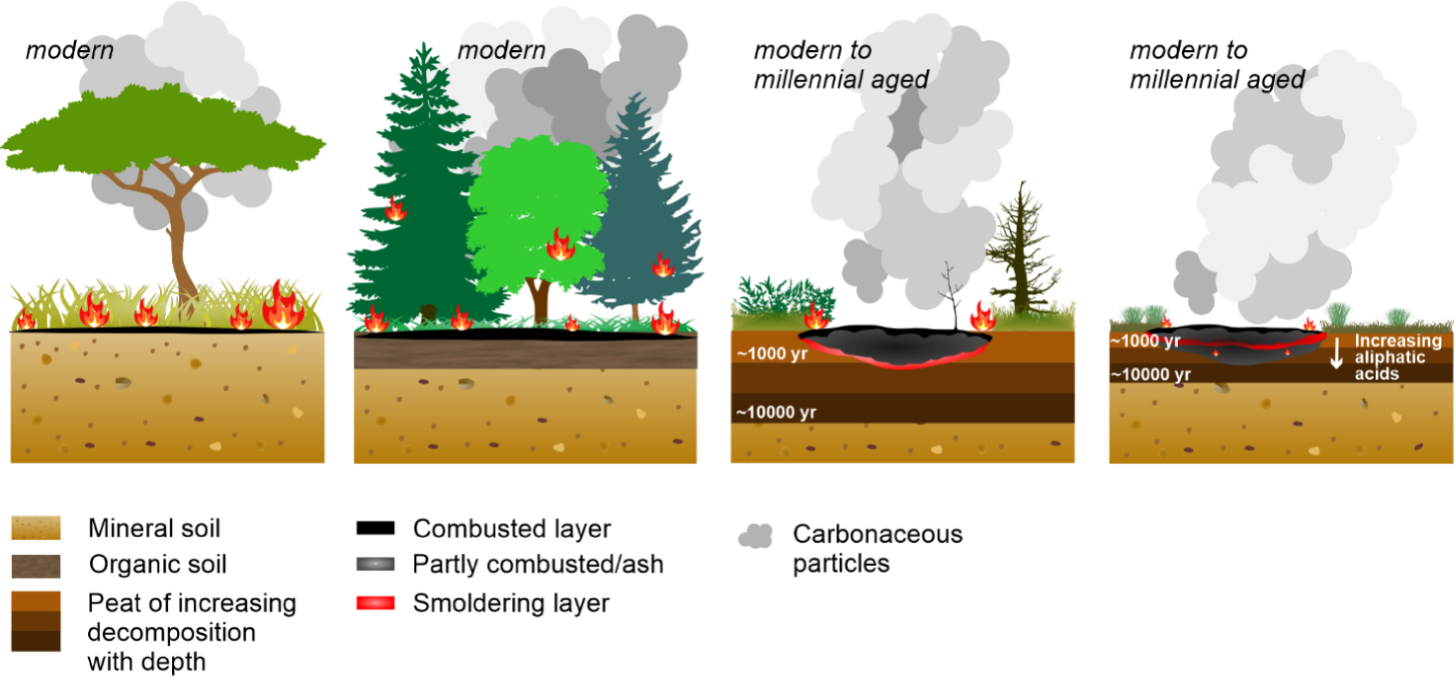
Diagram of burned material and age of carbonaceous particles released in savanna, boreal forest, boreal peatland, and Arctic peatland fires, presenting a conceptual interpretation of our combustion experiment results for natural wildfire scenarios. The flames, and combusted and smoldering layers, indicate at which depths the respective fires most likely consume organic material. The burn depth in both boreal and Arctic peat fires is ca. 20 cm,^51^ but the material is older in Arctic peatlands at the respective depth due to the lower peat accumulation rate. The low decomposition degree in Arctic peats leads to a buildup of aliphatic acids, resulting in increased flammability with depth (provided a low enough moisture content of the peat). Consequently, although Arctic fires may reach older peat layers, the emitted carbonaceous particles may not be older than those from boreal peats, as the oldest Arctic layers may be primarily combusted by flaming, producing less particulates. The age and depth of boreal and Arctic peats are indicative.

### 3.3 Age of the Elemental Carbon (EC) Fraction of Particulate Matter

Biomass combustion experiments have shown that EC emissions occur mainly during flaming combustion, while the bulk of total particulate carbon is emitted primarily as OC during smoldering combustion.^43,60^ Thus, it could be expected that the age of the EC and TC fractions collected on the filters in our experiments may diverge, as flaming predominantly consumes modern surface vegetation, producing relatively high EC emissions, while smoldering consumes deeper and thereby older organic material, producing relatively high OC emissions. However, here, the age of the EC fraction resembles the age of the TC fraction and is significantly older than the original biomass for many samples (SPH, CPFa+b, CPS, FBL, SAV) (Figure 2, Table S4).

The old age of EC indicates that some of the older biomass in our samples with a significant internal age variation was combusted during the early flaming stage of the experiments. This EC portion was much smaller than the OC amount produced by smoldering (Table 1). The smaller proportion of isotopic fractionation observed in the stable carbon isotope values of EC compared to TC of these samples supports the inference that EC was indeed released in the early flaming stages of combustion, as discussed in the following section (Figure 4, Table S5). Only SPH exhibited the hypothesized combustion behavior, in which the age of the collected EC fraction is significantly younger than that of TC (Figure 2), but the results should be considered only indicative due to the high measurement uncertainty (Table S4). The samples where no age differences were observed between the original biomass and the produced particulates (CRX, SHR, BFS) were likely the youngest and most homogeneous in age when sampling. The potential reasons for the younger age of EC than the original biomass of the Russian Arctic permafrost peat (APR) have been discussed above in connection to TC.

**Figure 4:**
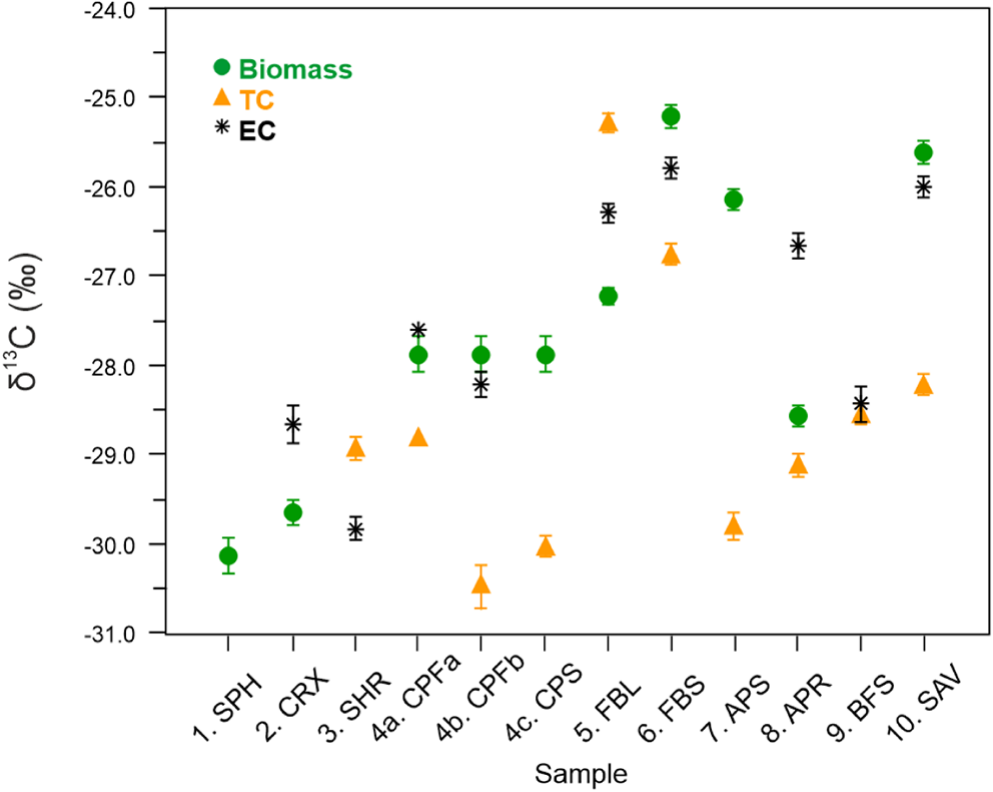
Stable carbon isotope (δ^13^C) values and uncertainty (standard deviation) for the original biomass and the particulate Total Carbon (TC) and Elemental Carbon (EC) fractions collected on filters during combustion.

### 3.4 Stable Carbon Isotope (δ^13^C) Values of Peat Combustion-Derived Carbonaceous Particles

The stable carbon isotope (δ^13^C) value for our peat biomass samples is on average −27.84‰ (*n* = 7, excluding nonpeat biomass BFS and SAV), varying between −25.23 and −30.13‰. These values exhibit no clear difference between peat collected from different climate conditions, nor compared to boreal forest surface or savanna samples (Figure 4, Table S5) and are within the range reported for other biomass in literature^43^ and specifically for surface peat from boreal and alpine environments.^71–74^

The ^13^C content of the TC collected on the combustion filters is depleted in comparison to the original biomass for CPFa+b, CPS, FBS, APR, and SAV (δ^13^C change from −27.04‰ to −28.68‰ on average), but is enriched for FBL (from −27.23‰ to −25.29‰) (Figure 4, Table S5). In contrast, the δ^13^C value of the EC fraction in these samples resembles the value of the original biomass. This was expected, as opposed to the smoldering-derived OC, EC is mainly emitted during the early flaming stages of combustion before significant isotopic fractionation occurs.^75–77^ However, the similar ages of EC and TC released during the combustion of samples CPFa, CPFb, CPS, and FBL, which are much older than the original biomass (Figure 2, Table S4), suggest that minor parts of the older material could combust in early flaming combustion phases for these samples. These findings confirm that OC and EC are produced from the combustion of different-aged parts of the mixed biomass samples of significant internal age variation and are in line with known δ^13^C fractionation during incomplete oxidation processes (see S6).

Importantly, our δ^13^C values for the peat combustion-derived TC and EC (−28.16 ± 1.6‰) are within the range previously reported for carbonaceous particles produced in biomass combustion, not including peat,^42–44,68^ presented in Figure 5. Thus, our peat combustion-derived δ^13^C values for EC and TC are not clearly distinguishable from the δ^13^C value (−26.7 ± 1.8‰) of biomass combustion-derived EC and OC. The latter value has been widely used for dual-carbon source apportionment of EC and OC in atmospheric and snow/ice samples.^38–40,42^ Our peat combustion-derived δ^13^C values are slightly more negative than the value generally used for biomass, but the difference is not significant enough to define a distinct δ^13^C range for peat combustion, especially considering the whole range of reported biomass combustion-derived particulate δ^13^C values (Figure 5). Thus, we conclude that carbonaceous particles derived from peat combustion cannot be separated from other biomass combustion-induced particles based on their δ^13^C values.

**Figure 5:**
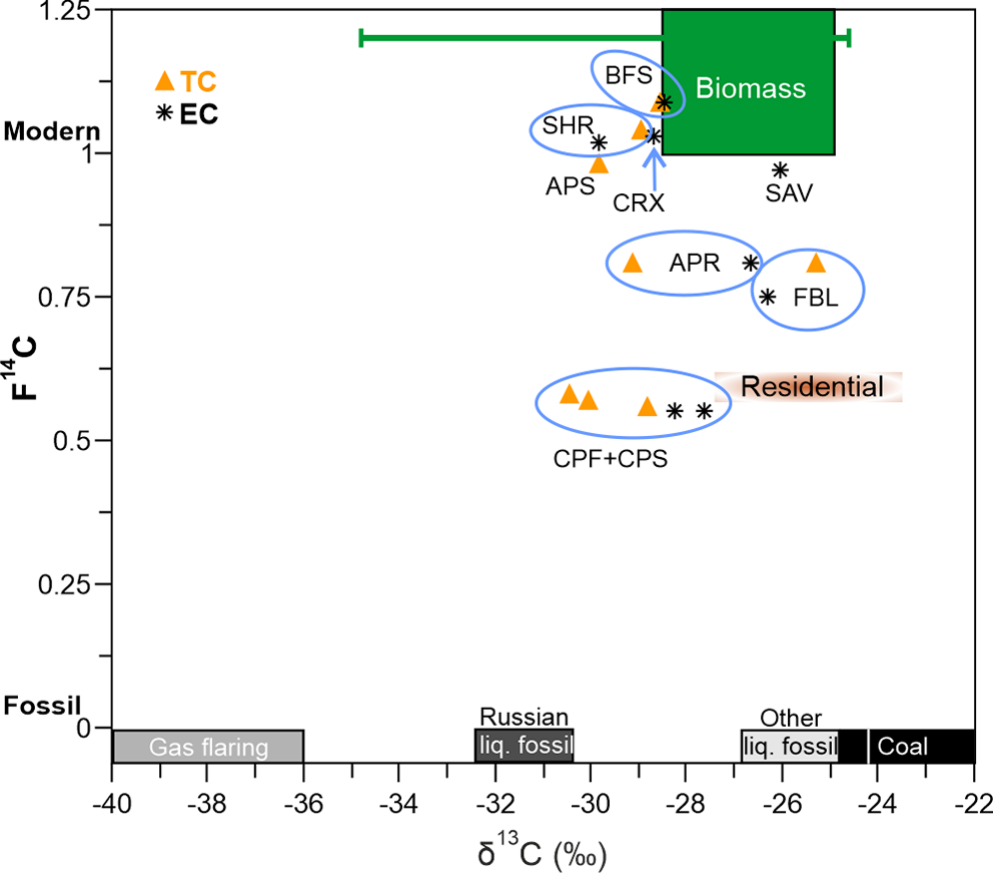
Cross plot of the stable carbon (δ^13^C) and radiocarbon (F^14^C) isotopic values of biomass combustion-derived carbonaceous particles, Elemental Carbon (EC), and Total Carbon (TC) in our samples. The expected δ^13^C and F^14^C ranges used in dual-carbon isotope source apportionment of OC and EC are shown as *rectangles* for biomass burning emissions (*green*), residential combustion (*light brown*), gas flaring emissions (*gray*), Russian and other (Chinese, Western European, and North American) liquid fossil fuel combustion (*dark and light gray*), and coal combustion (*black*), respectively, as estimated by Andersson et al.^42^ and Winiger et al.^40^ The *thin green bar* represents the range of reported δ^13^C values of biomass combustion-derived carbonaceous particles, based on which the expected range used in dual-carbon isotope source apportionment is established.^42^ The approximate position of residential combustion in the dimension is based on an estimate of its composition of 60% biomass, 39% coal, and 1% liquid fossil, as described by Huang et al.^78^ and Winiger et al.^40^

According to our results, smoldering Arctic and boreal peat fires can release millennia-aged carbon into the atmosphere, especially in the form of carbonaceous particles. The age of these particles is directly reflected in their radiocarbon signature, which consequently indicates a nonmodern source. At the same time, the δ^13^C value of the peat combustion-derived particles is similar to those of other biomass combustion-derived particles (Figure 5). Thus, peat burning interferes with dual-carbon isotope source apportionment of BC (and OC), i.e., skewing the result incorrectly toward a larger fossil fuel contribution (Figure 5), in areas where it is a significant source of carbonaceous particles, such as Indonesia,^48^ and especially the Arctic, where old carbon layers are present in combustible layers. The issue is further complicated, as the age of released carbonaceous particles is not necessarily proportional to the average age of the burned biomass. Without knowing the age (i.e., radiocarbon content) of carbonaceous particles or gases released in peat fires, it is impossible to correctly define these emissions present in natural aerosol or gas samples with the two-dimensional carbon isotopic source apportionment (Figure 5). Consequently, the potential error caused by peat burning must be considered in future dual-carbon isotope source apportionment studies on carbonaceous particles and carbon dioxide. For reliable source attribution of particulate carbon, particles produced from peat combustion need to be distinguished from other biomass burning particles through additional analytical methods, such as recently identified peat burning-specific organic compounds, which are stable in the atmosphere, and are not emitted in other wildfires.^63^

### 3.5 Light-Absorbing Properties of Peat Combustion-Derived Particles

The light absorption of the collected particles varies with the combustion conditions and intrinsic characteristics of the burned material. For instance, while the combustion of commercial peat seemed not to produce detectable BrC during flaming-dominated combustion (CPFa and CPFb), it produced substantial amounts of light absorption by BrC in the smoldering-dominated combustion (CPS) (Figure 6) highlighting the influence of burning conditions on the optical properties of the produced particles.

**Figure 6:**
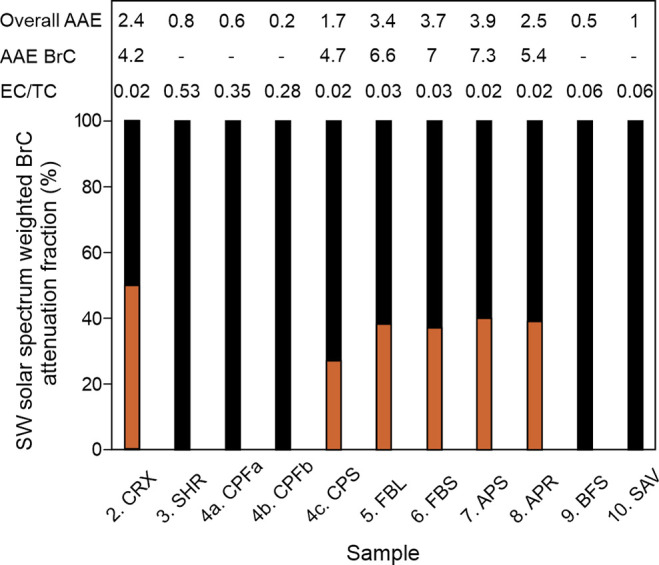
Short-wave solar spectrum-weighted BrC attenuation fraction (%) and Absorption Angstrom Exponent (AAE) for the overall sample and BrC of the combustion experiment filter samples quantified with the DRI 2015 Series 2 OCEC analyzer.^55,79,80^ Black bars represent light absorption by BC and brown bars by BrC. Also shown is the EC/TC ratio of the filter samples, with high values indicating flaming and low values indicating smoldering combustion conditions.

Significant amounts of BrC were detected in the filter samples produced during the smoldering-dominated combustion of different peats. BrC caused ca. 40–50% of the light absorption of the boreal and arctic peat (CRX, FBL, FBS, APS, APR) filters and ca. 27% of the commercial peat filter from smoldering combustion (CPS). Thus, below-ground organic material, namely, peat at various stages of decomposition and modern sedges (wetland vegetation), seems to be prone to smoldering combustion, yielding substantial amounts of BrC. The produced BrC absorbs light at a magnitude comparable to EC when weighed over the short-wave solar spectrum in these samples. These filters are brownish in coloration (Figure S5) and have the lowest EC/TC ratio of 0.02 to 0.03 (Figure 6, Table 1). The Absorption Ångström Exponent (AAE) of the observed BrC varies between 4.2 and 7.3, which is well within the range previously reported for BrC ranging from 1.5 to 12.^23,31,81^ The fuel type (e.g., wood, grass, or peat) influences the light-absorbing properties of OC released during their combustion, with carbonaceous particles generated from boreal peat burning having a higher AAE than biomass combustion particles in general.^22,23^ This supports our finding of comparably lower overall AAE for the SHR, BFS, and SAV samples (Figure 6) that contained predominantly wood, leaves, and litter, compared to the rest of our other samples consisting of peat (or wetland sedges (CRX)).

In the meantime, the particle light absorption is clearly dominated by EC for SHR, BFS, and SAV, as all the light absorption is attributed to EC by the analytical method used (Figure 6). SHR was produced primarily under flaming conditions and has the highest EC/TC ratio of all the samples (0.53; Figure 6). In contrast, BFS and SAV were produced primarily under smoldering conditions, although a short flaming phase was included in the PM collection of all filters. The EC/TC ratio of BFS and SAV is the highest among the smoldering samples (0.06; Figure 6). As EC is attributed to being solely responsible for the light absorption on the filters, the particulate OC produced in the combustion of these samples is, based on the DRI measurements, suggested not to significantly absorb light in comparison to the captured EC. Consequently, it would seem an intrinsic feature of these above-ground wood- and shrub-containing samples (SHR, BFS, SAV) not to produce light-absorbing OC, i.e., BrC, in significant amounts compared to EC even under smoldering conditions. However, recent studies have underscored the complexity to separate BC from darkest BrC that is optically more similar to BC than the lighter BrC,^82^ thus having properties intermediate of BC and BrC.^83^ The potential presence of this dark BrC may incorrectly be attributed to EC with the available methodology (DRI instrument), as flaming biomass combustion emissions and wildfire plumes have been reported to contain dominant amounts of dark BrC particles.^29^ Moreover, Samples 4c-10 were analyzed for BrC with other methodologies in Mukherjee et al.,^84^ showing a significant light absorption by BrC in all these samples, with flaming burning and woody material-containing samples generally producing darker BrC than peats and smoldering combustion. Consequently, it seems that the used DRI instrument may distinguish lighter-colored BrC more reliably than dark BrC^82^ and cause an overestimation of the fraction of light absorption by EC for our samples. Nonetheless, our results support the inference that the optical properties of combustion-derived particles vary under different combustion conditions depending on the combusted material.

Our BrC data can be further investigated in combination with the age of the combusted material and carbonaceous particles produced. We show that carbonaceous particles are primarily produced from older materials during the combustion of heterogeneously aged biomass. Further, combustion of below-surface older material is accompanied by a higher proportion of light absorption being caused by BrC compared to EC, as for boreal peat samples CPFa, CPFb, CPS, FBL (and likely FBS) (Figure 6). As an exception, combustion of modern CRX produced a significant BrC contribution to light absorption, likely as an intrinsic feature of the wetland-originated material resisting flaming combustion. Also, the combustion of hundreds to thousands of years-old below-surface Arctic permafrost peat (APS, APR) caused significant light absorption by BrC compared to EC on the filters (Figure 6), although some of the older peat layers combusted flamingly due to the high aliphatic compound content of the samples. In contrast, no BrC was detected on filters produced during the combustion of above-surface modern woody material (SHR, BFS, SAV; Figure 6). Consequently, the age of combusted biomass would be relevant to consider in future assessments of combustion product properties.

Our results underline the high BrC emissions from older-aged peat combustion, both under smoldering and flaming conditions, depending on its chemical composition. BrC undergoes complex chemical transformation and coating that causes its light-absorption properties to evolve during atmospheric aging,^29,31,85^ which is not captured by our filter samples and is beyond the scope of our study. However, BrC has been estimated to exert a direct radiative forcing accounting for 27–70% of BC forcing,^31^ and a non-BC light-absorbing component has been estimated to contribute to 50–75% of the total absorption of wildfire plumes.^29^ These values are similar to our estimation of the light-absorption fractions of BrC captured on the filters from smoldering peat combustion.

### 3.6 Climate Implications of Carbonaceous Particles Released in Peat Fires

With the northward extension of the wildfire regime, an inevitable shift is occurring from flaming burning of above-ground biomass to smoldering burning dominated by below-ground biomass. This results in more incomplete combustion and larger emissions of particulate carbon per unit biomass.^14^ Savanna and boreal forest fires have been an integral part of these ecosystems, where the carbon released in recurring fires tends to be quickly taken up again by vegetation regrowth.^14,17,86^ However, the increasing fire frequency and deep smoldering in the Arctic-boreal biome have led to concerns about ancient carbon, stored under stable conditions for millennia outside the active carbon cycle, being released into the atmosphere, thereby perturbing the global carbon cycle and accelerating climate warming beyond current estimates.^4,7,17,87,88^ In Alaska, radiocarbon dating has shown that carbon released in tundra fires has been only about 50 years old,^10,11^ suggesting no ancient carbon release. Meanwhile, in Indonesia, the radiocarbon content of smoke originating from peat fires corresponded to a carbon pool of combusted organic matter with a mean turnover time of 800 ± 420 years, with gradually deeper and older carbon projected to be released in future fires.^48^ Our results suggest that millennial-aged particulate and gaseous carbon, far surpassing the age of modern (less than ca. 100 years old) carbon typically cycling in the centennial-scale fire-driven boreal forest carbon cycle,^86^ may also be released from Arctic tundra and peat fires and from fuel peat combustion. Such release of ancient carbon causes long-lasting feedback into the global climate system, as semifossil carbon is added into the atmosphere. Thus, additional field observations on the age of carbon released in Arctic and boreal wildfires are required to comprehensively assess their role in the global carbon cycle.

In addition to the potential release of millennial-age carbon from previously stable carbon reservoirs, smoldering high-latitude fires may be consequential for the global carbon cycle due to their substantially higher carbonaceous particle emissions compared to flaming boreal, and particularly savanna, fires. Current BC and PM emissions from wildfires above 65 °N exceed those from the biggest anthropogenic sources of the region.^89^ In our experiment, smoldering peat combustion produced substantial emissions of light-absorbing BrC in addition to EC, and EC yields were non-negligible during smoldering combustion, in contrast to previous findings for boreal peat combustion.^23^ Our measurements of peat-combustion-derived particles suggest that these do not exhibit a source-specific carbon isotopic composition. Therefore, additional analytical methods, such as organic compound fingerprints,^63^ are required to distinguish them from particles originating from other biomass sources.

With a changing Arctic fire regime, Arctic fires and their particle emissions are underestimated in current emission inventories,^36,89^ while BrC is completely absent or poorly constrained as a distinct component in global scale climate models.^90,91^ Moreover, the climate impacts of ancient carbon emissions from Arctic-boreal fires are currently, to the best of our knowledge, not traced or considered in any climate model. Our results suggest that these deficiencies might cause severe underestimation of the climate impact of Arctic and boreal fires on ongoing climate warming, particularly within regions of snow- and ice-covered surfaces that are prone to enhanced melting following particle deposition and albedo reduction.^27,37,92^ Moreover, in the Arctic, low-level clouds are common, and wildfire plumes often reside above them. This positioning leads to enhanced solar absorption and positive radiative forcing within the atmosphere.^36^ As smoldering Arctic and boreal wildfires are expected to increase due to climate warming and following changes in permafrost environments,^2,7,14,93–95^ they are likely to create a positive feedback to the global climate system via the release of millennial-aged carbon as gaseous and particulate compounds, including light-absorbing BrC.

## Supplementary Material



## Data Availability

The numerical measurement analyses data of carbonaceous particles and biomass and their respective isotopes are available at the Finnish Meteorological Institute repository METIS, provided by EUDAT under the CC BY 4.0 open-access license at https://doi.org/10.57707/fmi-b2share.v46vg-1ct03.

## Notes

Additional details on biomass samples, the experimental setup (including photographs and diagrams) for biomass combustion, and analysis methodologies, as well as supplementary result tables on dual-carbon isotope concentrations of the biomass and filter samples (PDF)
