# Supplementary material for: Arctic and Boreal Wildfires Impact Climate by Releasing Ancient Carbon and Light-Absorbing Particles
Source: Environ Sci Technol. 2026 Apr 9;60(15):11608–20. doi: 10.1021/acs.est.5c17130 (PMC13104179; doi:10.1021/acs.est.5c17130)
Supplement: Supplementary file 1 [file es5c17130_si_001.pdf]

SUPPORTING INFORMATION

Arctic and Boreal Wildfires Impact Climate by Releasing Ancient Carbon and Light-Absorbing Particles

Meri. M. Ruppel[1,2,], Markus. Somero[3], Olli Sippula[3,4], Mika Ihalainen[3], Juho Louhisalmi[3], Jarkko Tissari[3], Negar Haghipour[5,6], Johan Ström[7], Minna Väliranta[2], Kajar Köster[3], Ville Vakkari[1,8], Kerneels Jaars[8], Lin Huang[9], Rienk H. Smittenberg[10]*

[1]Atmospheric Composition Unit, Finnish Meteorological Institute, 00560 Helsinki, Finland

[2]Environmental Change Research Unit (ECRU), Department of Environmental Sciences, University of Helsinki, Helsinki FI-00014, Finland

[3]Department of Environmental and Biological Sciences, University of Eastern Finland, Kuopio FI-70211, Finland

[4]Department of Chemistry and Sustainable Technology, University of Eastern Finland, Joensuu FI-80101, Finland

[5]Department of Earth Sciences, Geological Institute, ETH Zurich, Zurich 8092, Switzerland

[6]Laboratory for Ion Beam Physics, ETH Zurich, Zurich 8093, Switzerland

[7]Department of Environmental Science (ACES), Stockholm University, Stockholm 11418, Sweden

[8]Atmospheric Chemistry Research Group, Chemical Resource Beneficiation, North-West University, Potchefstroom 2520, South Africa

[9]Climate Research Division, Atmospheric Science & Technology Directorate, Environment and Climate Change Canada, Toronto, ON M3H 5T4, Canada

[10]Swiss Federal Institute for Forest, Snow and Landscape Research WSL, Zuercherstr. 111, Birmensdorf 8903, Switzerland

* Corresponding author: meri.ruppel@fmi.fi

Description of Supporting Information:

Number of pages: 22
Number of tables: 5
Number of figures: 7

Content included:

Table S1: Detailed description on biomass sampling locations.

Text **S.1.** Biomass sample pretreatment, and determination of elemental composition and moisture content, including **Table S2** of these results.

Text S.2. Details on the experimental setup for biomass combustion, particulate matter filter collection, and gaseous emission measurements, including **Figures S1-S3** presenting photos and diagrams of the combustion setups, and **Figure S4**, a photo of particulate matter filters collected during the combustion experiments.

Text S.3. Calculation of combustion efficiency during the combustion experiments and particulate carbon emission factors

Text S.4. Details on the Total, organic carbon and elemental carbon (TC, OC and EC) quantification from filters by thermal-optical analysis, including **Table S3** on the heating and atmospheric condition steps during analysis by three different protocols, and **Figure S5** comparing acquired results by these protocols.

Text S.5. Description on the methodology for Brown carbon (BrC) analysis from filters

Text S.6. Description on the correction for isotopic fractionation in the carbon isotope determination of total and elemental carbon (TC and EC).

Text S.7. Description of age, plant composition and decomposition degree variation in peat profiles, including **Figure S6** of peat profiles collected at Alkhornet tundra, Svalbard, showing increasing degree of decomposition and age with depth, and **Figure S7** of vegetation composition changes over the last 2000 years in peat profiles collected at a permafrost peatland in Rogovaya, Russia.

Table S4. Radiocarbon ($\Delta^{14}C$) fraction modern and the age of original biomass samples and produced particulate matter on filters from the combustion experiments.

Table S5. Carbon isotope ($\delta^{13}C$) values for the original biomass and the particulate carbon fractions collected on filters during combustion

References

Table S1. Details on biomass samples and collection locations for this study.

Sample number	Sampling site/ Sample ID	Coordinates	Vegetation type	Sampling method	Sampling depth
**1**	Salamajärvi (Finland)<br>***Sphagnum*-peat**<br>**SPH**	63°16'N,<br>24°48'E	Northern boreal peatland, *Sphagnum*-dominated	Surface collected with a shovel	0-20 cm
**2**	Skállovárri (Finland)<br>***Carex*-peat**<br>**CRX**	69°49' N<br>27°12' E	Subarctic permafrost peatland,<br>sedge-dominated	Surface collected with a shovel	0-20 cm
**3**	Karlebotn (Norway)<br>**Shrub-peat**<br>**SHR**	70°07'38.8"N<br>28°33'06.0"E	Subarctic permafrost peatland, shrub-dominated	Surface collected with a shovel	0-20 cm
**4**	Petronneva, (Finland)<br>**Commercial peat (CP)**<br>**CPFa+b, CPS**	62°58'13"N<br>26°58'36"E	Boreal peatland	Unknown	Unknown
**5**	Orivesi (Finland)<br>**Finnish boreal peat (Lakkasuo)**<br>**FBL**	61°47'21.6"N<br>24°18'35.9"E	Boreal peatland, *Sphagnum*-dominated	Peat box corer,<br>peat profile	0-30 cm
**6**	Pirkanmaa (Finland)<br>**Finnish boreal peat (Siikaneva)**<br>**FBS**	61°49'28.0"N<br>24°08'25.8"E	Boreal peatland, *Sphagnum*-dominated	Peat box corer,<br>peat profile	0-30 cm
**7**	Alkhornet (Svalbard)<br>**Arctic permafrost peat (Svalbard)**<br>**APS**	78°12'48.7"N<br>13°49'36.1"E	Permafrost peatland, herbaceous moss tundra	Peat box corer,<br>peat profile	0-30 cm
**8**	Rogovaya (Komi Republic, Russia)<br>**Arctic permafrost peat (Russia)**<br>**APR**	67°19'43.0"N<br>62°36'18.1"E	Permafrost peatland, sedge-dominated	Peat box corer,<br>peat profile	0-50 cm
**9**	Evo (Finland)<br>**Boreal forest surface**<br>**BFS**	61°11'48.8"N<br>25°05'39.2"E	Boreal forest surface (vegetation, litter and soil organic layer)	Collected from the surface with a shovel	Surface
**10**	Welgegund (South Africa)<br>**Savanna**<br>**SAV**	26°34'12.5"S<br>26°56'21.5"E	Woody plant material from savanna; branches of: *Celtis Africana, Searsia pyroides, Vachellia karroo, Ziziphus mucronate, Asparagus laricinus, Gymnosporia buxifolia, Vachellia erioloba, Euclea undulata, Senegallia caffra, Pavetta zeyheri, Vangueria infausta and Zanthoxylum capens*	Collected above surface	Above surface

S.1. Biomass sample pretreatment, and determination of elemental composition and moisture content

The original biomass samples 1-3 and 5-9 were dried at 60°C for 24-72 hours in a ventilated drying oven, resulting in an approximate 10 % (or lower) moisture content for the samples. Following drying, the samples were most likely drier than during a natural wildfire, but this approach was chosen as: 1) A similar moisture content between the samples ensures the repeatability of the results, i.e. confirms that possibly acquired differences in particulate matter emissions between samples are not caused by different moisture contents, and 2) our results are comparable to previous combustion experiments performed on Arctic and boreal peats with same procedures in the same laboratory[1,2] and elsewhere[3]. After drying, the samples were not homogenized as this could have altered the physical composition, such as the grain size of the material, which could affect its combustion behavior. Consequently, potential subsamples for experiments were carefully collected over the full vertical length/depth of each sample to secure same composition (e.g. in age) for each subsample. The commercial peat (Sample 4) was received as dried and homogenized fuel briquettes, and the savanna tree sample (Sample 9 SAV) was received and studied as collected from the field.

The carbon and nitrogen content of the dried biomass samples 1-3 and 5-8 was determined with a Flash 2000 Organic Elemental Analyser (Thermo Fisher) after homogenizing a mg-sized subsample with a stainless-steel ball grinder. The composition (C/N/S/H %) and moisture content of Samples 4, 9 and 10 were determined commercially from a subsample by the Eurofins Environment Testing Finland Oy using European-wide accredited analytical procedures. The results are presented in Table S2. The determined moisture content of 8.2 to 9.7 % of Samples 4, 9 and 10, and particularly of sample 9 which was dried in the same way as our samples 1-3 and 5-8, supports the estimated moisture content of approximately 10 % or less for the samples 1-3 and 5-8.

Table S2. Composition (carbon, nitrogen, sulfur and hydrogen %) and moisture content (%) of fuel biomasses, and the combusted biomass amount in individual combustion experiments.

**Sample ID**	**Carbon content (%)**	**N/S/H content (%)**	**Moisture content (%)**	**Combusted amount**
**1.** *Sphagnum* peat (**SPH**)	46.7	*na*	~ 10*	~ 3 kg
**2**. *Carex* peat (**CRX**)	48	*na*	~ 10*	~ 3 kg
**3.** Shrub peat (**SHR**)	*na*	*na*	~ 10*	~ 3 kg
**4a.** Commercial peat (**CPFa**)	57	1.92/0.2/5.8	9.7	~ 3 kg
**4b.** Commercial peat (**CPFb**)	57	1.92/0.2/5.8	9.7	~ 3 kg
**4c.** Commercial peat (**CPS)**	57	1.92/0.2/5.8	9.7	50 g
**5.** Finnish boreal peat (Lakkasuo) (**FBL**)	47	-/*na*/*na*	~ 10*	50 g
**6.** Finnish boreal peat (Siikaneva) (**FBS**)	47.6	-/na/na	~ 10*	50 g
**7.** Arctic permafrost peat (Svalbard) (**APS**)	27.9	1.85/*na*/*na*	~ 10*	50 g
**8.** Arctic permafrost peat (Russia) **(APR**)	51.7	2.9/*na*/*na*	~ 10*	50 g
**9.** Boreal forest surface (**BFS**)	50.5	1.19/0.11/5.45	8.2	350 g
**10.** Savanna (**SAV**)	48.8	0.88/0.05/5.6	9.4	60 g

**Estimated approximate moisture content*

S.2. Details on the experimental setup for biomass combustion, particulate matter filter collection, and gaseous emission measurements

Flaming and smoldering combustion are tightly related, and during natural wildfires one combustion mode can transition to the other[5]. Typically, a boreal forest fire may be ignited by lightning and move fast by high-temperature flaming surface vegetation burning, while flameless, low-temperature smoldering of thicker fuels (on and below-ground) is sustained for days after the flaming wildfire has passed[5]. The majority of carbon loss of a boreal forest fire originates from combustion of the soil organic layer[6].

In this study, we aim to investigate the isotopic composition and light-absorption of carbonaceous particles released in flaming and smoldering-dominated combustion. Variation in the characteristics of the produced particles could relate both to the different combustion conditions (e.g. temperature) in flaming vs. smoldering conditions, and the material's natural propensity to burn preferentially either by flaming or smoldering (e.g. surface vegetation under flaming conditions and soil material in smoldering conditions[5,7], or different plant species having natural propensities[8]). As both combustion modes occur in natural wildfires and often

inseparably, we chose to study flaming- and smoldering-*dominated* combustion, including both combustion modes in the Particulate Matter (PM) sampling period but in different ratio, instead of strictly collecting PM samples of either flaming or smoldering on the filters.

The biomass samples were combusted to produce PM collected on filters in two separate setups: 1) Flaming-dominated experiment for samples SPH, CRX, SHR, and CPFa+b; and 2) Smoldering-dominated experiment for samples CPS, FBL, FBS, APS, APR, BFS, and SAV. Combustion was performed at the University of Eastern Finland (UEF), Fine Particle and Aerosol Technology Laboratory (https://sites.uef.fi/fine/front-page/simo/ and www.uef.fi/ilmari). The first combustion experiment was conducted at SIMO (Residential Wood Combustion Simulator), where the peat was combusted in a closed combustion chamber (with open air intake, and air to fuel ratios varying between 4.2 and 12.4 indicating oxygen-rich combustion), leading to relatively high combustion temperature and *primarily* flaming combustion, with less prevalent smoldering phases. As in natural wildfires the surface vegetation is most susceptible to flaming combustion[5,9,10], we performed the flaming-dominated experiment on our surface peats from the top 15-20 organic material containing mainly young and living material, and for comparison also from the commercial peat collected at unknown depth (Table S1). In contrast, the second experiment conducted at the ILMARI (Aerosol physics, chemistry and toxicology research unit) represented open biomass burning, with high excess air ratios and consequent low combustion temperatures, which therefore resulted in *primarily* smoldering conditions mimicking natural (northern) wildfires, but also included short flaming phases. The smoldering-dominated experiment was performed for peat collected at deeper (and thereby expectedly older) soil depths (0-30 and 0-50 cm) (Table S1) that would most likely smolder in natural wildfires[5,9,10], and compared it again with commercial peat and biomass from boreal forest surface and savanna environments. In both experiments, the PM was collected on the filters for the full duration of the combustion cycle, including the initial smoldering, a short or intentionally extended (in the first experiment) flaming phase, and residual smoldering. Section S.3. describes how the combustion efficiency, i.e. flaming vs. smoldering conditions, were verified during the experiments.

For the flaming combustion experiment, the complete (~ 3kg dry mass (Table S2)) surface peat samples (SPH, CRX, SHR) and ~3kg of commercial briquettes (CPF) were placed in a stainless-steel tray, originally

designed as a so-called wood pellet burner basket for wood stoves (Fig. S1a). The burner tray made of a perforated plate allowed combustion air to enter the fuel bed. The burner tray was placed on a grate of a wood stove, and the peat fuel was ignited by burning birch wood sticks below the grate in the ash box of the stove, allowing the flames to heat the peat samples. Upon ignition of the peat and before commencing the filter sampling period, the flaming birch wood was removed from the stove to avoid contamination of the PM samples with wood smoke. With this setup, high temperature, primarily flaming, combustion was achieved. The exact burning temperature and the dominance of flaming vs. smoldering combustions were not well controlled and varied between the samples, mainly due to the intrinsic nature of each biomass sample

For this flaming experiment, combustion times in each full combustion cycle ranged between 10 and 25 minutes. PM was collected from the combustion exhaust smoke over the full combustion on pre-baked quartz fiber filters in two different ways. For samples SPH, CRX and SHR, the PM was collected onto 90 mm filters from diluted smoke using a 10 µm cyclone (Fig. S2a). In contrast, PM of samples CPFa and CPFb was collected on two parallel 47 mm filters from diluted smoke that was run first through a 10 µm cyclone and then a 2.5 µm impactor, resulting in a 2.5 µm size-cut for these samples (Fig. S2b). Organic gaseous carbon (OGC) was measured in both measurement setups with a flame ionization detector (FID) analyzer (FIDAMAT 6, Siemens AG, Nürnberg, Germany). Carbon dioxide (CO_2) and carbon monoxide (CO) concentrations were measured with single gas analyzers (ULTRAMAT 23, Siemens AG, Nürnberg, Germany). The gases were measured from the flue gas after passing a ceramic filter to remove particles from the samples (Fig. S2). The dilution ratio was calculated as described in Tissari et al.[11].

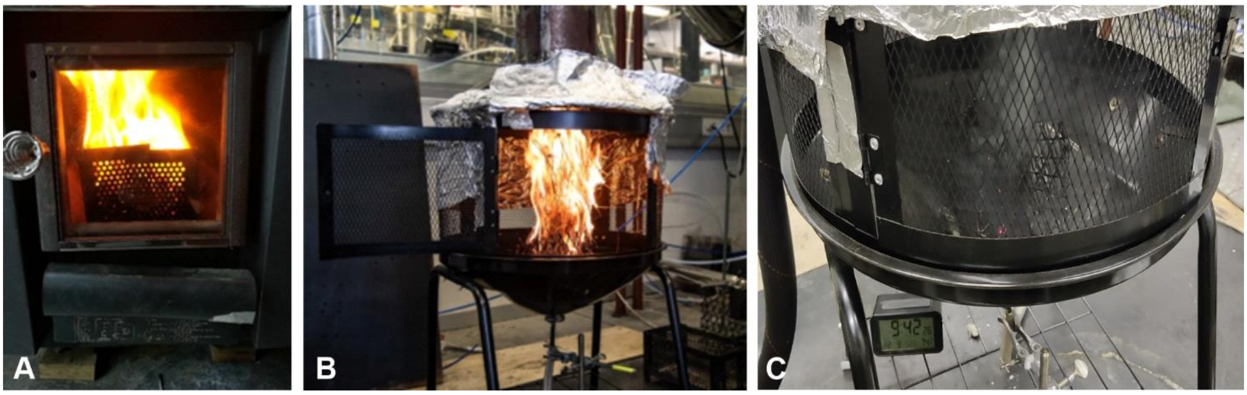

Figure S1. Photos of combustion experiment setups. A) Closed combustion chamber used for the flaming combustion experiment of samples SPH, CRX, SHR, CPFa and CPFb. B) Open combustion experiment setup for smoldering combustion of samples CPS, FBL, FBS, APS, APR, BFS and SAV. The photo shows flaming combustion of a SAV sample (not for this study). C) Close-up of smoldering combustion of sample APS.

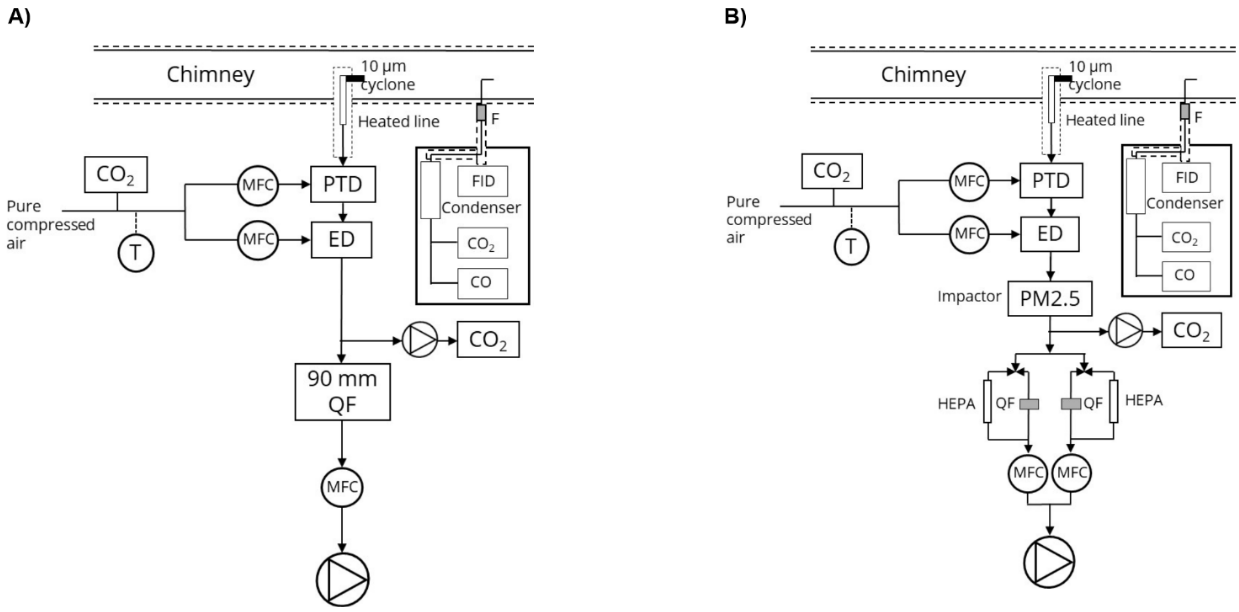

Figure S2. Setup for particulate matter collection on quartz fibre filters (QF) and measurement of gases during the flaming-dominated combustion experiment. A) Combustion experiments performed for samples SPH, CRX and SHR. B) Combustion experiments performed for samples CPFa and CPFb. The smoke sample arrives through the chimney and first passes the 10 µm cyclone before entering the particle sampling line. The carbonaceous gases are measured from the flue gas after passing a ceramic filter (F). PTD = Porous Tube Diluter; ED = Ejector Diluter; MFC = Mass Flow Controller; PM2.5 = Impactor with a particle cut off size of 2.5 µm; FID = Flame Ionization Detector, HEPA = High Efficiency Particulate Air filter.

In the second combustion experiment, a setup constructed for open firing, simulating smoldering open burning of peatlands in arctic and boreal environments[5], was utilized to combust the samples CPS, FBL, FBS, APS, APR, BFS, and SAV. A 50 g subsample of each dried and lightly mixed peat sample (FBL, FBS, APS, APR), 60 g of the savanna sample (SAV), and ca. 350 g of the boreal forest surface sample (BFS) were inserted in a steel cage, which was placed on a concave plate surrounded by metal mesh walls (Fig. S1b, c). A hood above the samples was connected to a chimney equipped with a flue gas fan, which drew the exhaust gases and particles. The biomass samples were placed around a heating element that ignited the samples. The heating element was supplied with constant power and was deactivated either immediately after visible flames were observed or one minute after ignition to ensure the flame did not extinguish. During heating, the biomass initially began to smolder before fully igniting in flames. The ignition process took 6 min for the commercial peat, 4 min for the Svalbard permafrost peat (APS) and 3 min for other samples, as described in more detail in Schneider et al.[1].

PM samples were collected in the smoldering experiment from the exhaust smoke on pre-baked quartz fiber filters (QFF, Pallflex Tissuquartz) for 12–67 min (depending on the duration of the full combustion from the beginning of the initial smoldering, short flaming and to the end of the residual smoldering) with a flow rate of 90 l min^{-1} (Fig. S3)[1]. Each biomass sample was ignited the same way, and thus the combustion behavior and the resulting emissions are an intrinsic property of the respective samples[1]. The produced PM filters (Fig. S4) were kept frozen until analyses.

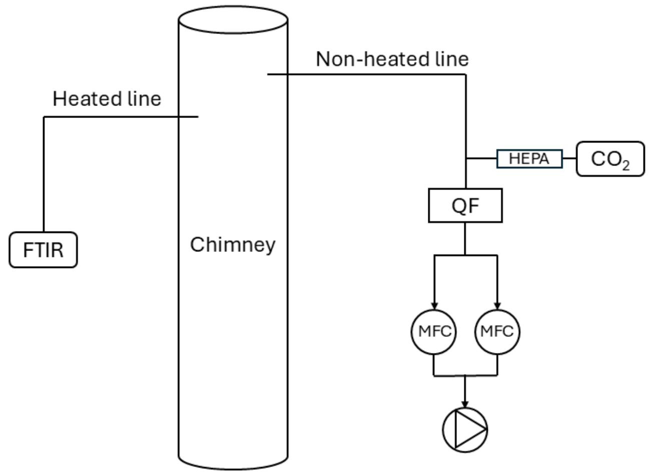

Figure S3. Setup for particulate matter collection on quartz fibre filters (QF) and gas phase measurements during the smoldering-dominated combustion experiment for samples CPS, FBL, FBS, APS, APR, BFS, SAV. The smoke sample arrives through the chimney and enters a non-heated sampling line without a cyclone or impactor. The heated line for FTIR is set to 180 °C. MFC = Mass Flow Controller, FTIR = Fourier-Transform Infrared Spectroscopy, HEPA = High Efficiency Particulate Air filter.

In the smoldering-dominated combustion experiment the gaseous compounds were measured from the fresh, undiluted biomass burning emissions using an online multicomponent FTIR analyzer (FTIR DX4000, Gasmet Technologies Inc.) and single gas CO_2-analyser (ULTRAMAT 23, Siemens AG, Nürnberg, Germany), except for SAV, which didn't have Siemens ULTRAMAT 23 available for it (Fig. S3).

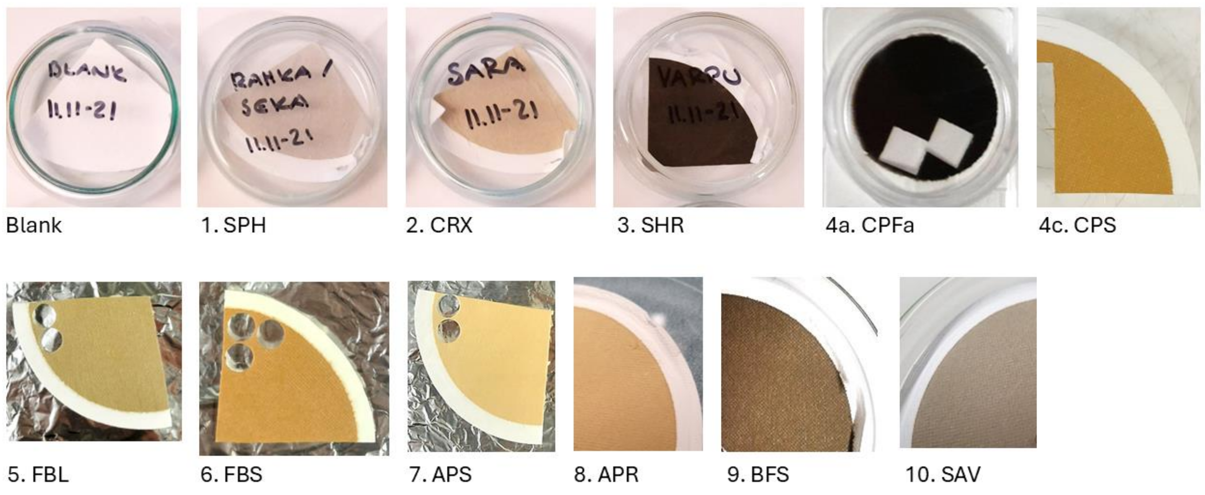

Figure S4. Photographs of produced PM filter samples. Photos by Meri Ruppel and Viljami Luostari.

S.3. Calculation of combustion efficiency during the combustion experiments and particulate carbon emission factors

Quantification of carbonaceous gases and particles during the combustion experiments permitted the calculation of the combustion efficiency and emission factors of produced particle matter. A common parameter to characterize burning efficiency during biomass burning experiments is the modified combustion efficiency (MCE), which describes the share of carbon dioxide to the sum of CO and CO_2 emissions[12]. We calculated MCEs from the average increase in CO_2 and CO concentrations during the complete sampled combustion period of each experiment relative to the background concentrations as described in detail in Mukherjee et al.[2] Experimental burns with average MCE values smaller than 0.9 were defined as smoldering-dominated combustion, while the combustions with average MCE values larger than 0.9 were classified as flaming dominated (Table 1).

Emission factors of the carbonaceous particles produced in the combustion experiments were calculated by the carbon mass balance method outlined in Yokelson et al.[13] considering the carbon fraction in the combusted sample and the total carbon released during the combustion (as both gases and particles)[2]. The particulate matter yields, i.e. total, organic and elemental carbon (TC, OC and EC) concentrations, were quantified by OCEC analyses of the collected filters, as outlined in S.4.

S.4. Total, organic carbon and elemental carbon (TC, OC, and EC) quantification from filters by thermal-optical analysis

The concentrations of the biomass combustion-derived carbonaceous particles on the PM filters were determined using a thermal-optical carbon analyzer (Sunset Instrument (Model 5L)[14]), where the carbonaceous aerosol fractions are separately quantified based on their temperature-specific volatilization, by controlled temperature and redox conditions, and optical correction for pyrolytically generated carbon

(charring)[14]. In the first several stages of the analysis, a filter punch is heated stepwise in a helium atmosphere, releasing organic carbon (OC), carbonate carbon (CC) and potentially pyrolyzed organic carbon (PyOC). In the last few stages, Elemental Carbon (EC, a thermal optical proxy for BC) is released by heating the filter stepwise in an oxygen-helium atmosphere. The carbon fractions are released from the filter in the form of carbon dioxide, which is then reduced to methane for quantitative detection by a flame ionization detector[14]. During analysis, the transmittance of the filter is monitored using laser light (wavelength 678 nm), allowing for optical correction of charring, i.e. potential pyrolysis of OC to EC during the analysis[15].

Here, the ECT9-protocol, recently developed for the collection of OC and EC fractions to determine the carbon isotopic composition of filter samples[16], was used. Following the ECT9 protocol, the carbon fractions are released in three steps: (1) OC at 550 °C for 600 s in pure Helium, (2) PyOC and CC at 870 °C for 600 s in pure Helium, and (3) EC at 900 °C for 420 s in a mixture of 2% oxygen with 98% Helium. The ECT9 protocol is specifically developed to volatilize all potentially pyrolyzed OC from the filter before the commencement of the oxidizing phase quantifying EC. According to extensive testing, this facilitates a clear-cut separation of the released OC and EC for their isotope analyses[16]. Table S3 lists the heating steps of the ECT9 protocol in comparison to the standard EUSAAR_2 protocol[15] used in Europe, and the IMPROVE_A protocol[17] widely used in the U.S. Chemical Speciation Network. The TC, OC and EC concentrations obtained for four of our samples (BFS, FBL, FBS, SAV) with these three protocols show comparable results (Fig. S5), which indicates that also the OC and EC emission factors calculated based on ECT9 measurements for this study (SPH, CRX, SHR, CPFa, CPFb) and IMPROVE_A measurements published in Mukherjee et al.[2] (CPS, FBL, FBS, APS, APR, BFS, SAV) are comparable.

Table S3. Temperature and atmosphere conditions in the EUSAAR_2, ECT9 and IMPROVE _A protocols during thermal-optical measurements both in the Sunset and DRI 2015 Series 2 OCEC analyzers.

Step	EUSAAR_2	ECT9	IMPROVE_A
	T (° C), duration (sec)	T (° C), duration (sec)	T (° C), duration (sec)
He 1	200, 120	550, 600	140, 140-580
He 2	300, 150	870, 600	280, 140-580
He 3	450, 180		480, 140-580
He 4	650, 180		580, 140-580
He/O_2 1	500, 120	900, 420	580, 140-580
He/O_2 2	550, 120		740, 140-580
He/O_2 3	700, 70		840, 140-580
He/O_2 4	850, 80		

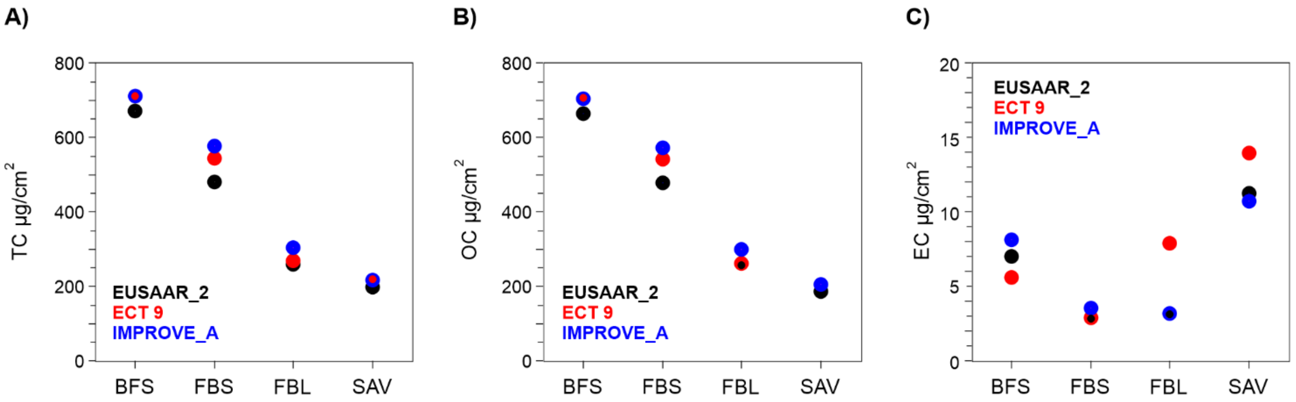

Figure S5. Comparison of total, organic and elemental carbon (TC, OC and EC) concentrations (µg/cm^2) for the boreal forest surface (BFS), Finnish boreal peat from Lakkasuo (FBL) and Siikaneva (FBS) and savanna (SAV) samples obtained with the Sunset OCEC analyzer for the EUSAAR_2, ECT9 and IMPROVE_A temperature protocols (Table S3). Note, that the analyzed filters may be heterogenic in their particle loading which may account for some of the differences.

S.5. Brown carbon (BrC) analysis from filters

Light-absorbing organic carbon, i.e. Brown Carbon (BrC), was estimated on the filters with a DRI 2015 Series 2 Multi-Wavelength Thermal/Optical Carbon Analyzer (Aerosol Magee Scientific) using the Eusaar_2 temperature protocol (Table S3). The DRI instrument operates similarly to the Sunset instrument, but it monitors the optical transmittance and reflectance of the sample simultaneously at 7 wavelengths (405, 445, 532, 635, 780, 808, and 980 nm), which uniquely allows BrC quantification from the OC fraction[18]. The contribution of BrC to the optical attenuation measured by the instrument is calculated by the DRI instrument software by assuming an Absorption Ångström Exponent (AAE) of 1 for BC (although also lower values have been reported in literature[7]), as explained in the procedure described in Chow et al.[19,20]. Here, we assume that this AAE value is also valid for EC, which makes BC and EC interchangeable. Thus, any excess attenuation is attributed to BrC. The calculated overall AAE of the measured samples was based on a fit to the attenuation values over the seven wavelengths of the instrument between 405 and 980 nm.

As BrC is a complex mixture of different materials, its light absorption properties vary extensively, for instance, according to its formation temperature[21–23]. Thus, it cannot be directly quantified as mass with the DRI instrument. However, the excess attenuation recorded by the instrument is spectrally dependent, and the fraction of the light absorption on the filter between EC and BrC can be expressed as percentages. To calculate a value for each fitted spectra, values were weighed with factors approximating the solar spectrum, i.e. 0.137, 0.207, 0.182, 0.156, 0.131, 0.106, and 0.081 for wavelengths 400, 500, 600, 700, 900, and 1000 nm, respectively. A value of zero BrC does not necessarily mean that BrC is absent, but rather that the contribution to the attenuation is too small to be separated from BC using the DRI analytical method. Hence, for these cases, all the attenuation can be explained by the BC model of using the unit Angstrom exponent, and the relative contribution by BrC (or anything else with an optical property different from BC) is comparably small when all the attenuation can be explained by the unit Angstrom exponent. The newly identified existence of so-called dark-BrC which is abundantly present in wildfire plumes and has similar optical and other properties to BC[21,23,24], may potentially be misallocated to BC with the current methodology due to its optical properties closely reminding those of BC, but further discussion of the issue is beyond the scope of our study.

S.6. Correction for isotopic fractionation in the carbon isotope determination of total and elemental carbon (TC and EC)

Combustion processes are accompanied by $\delta^{13}C$ fractionation when the oxidation process is incomplete, such as in our combustion experiment, that produced PM and CO in addition to CO_2. $\delta^{13}C$ fractionation can vary from nearly zero to highly positive or negative values. Its magnitude depends on combustion conditions, and its direction varies in an open combustion system[25–27] such as ours. Combustion-related isotopic fractionation is expected to occur between different parts of non-homogenous fuels, as different material 1) possesses different isotopic composition (e.g. above-ground vs. below-ground material), and 2) burns at different temperatures and in different combustion phases (flaming vs. smoldering)[28]. No corrections were performed when reporting isotope results, and fractionation occurred in our combustion experiment. Correction for isotopic fractionation occurred during the analytical procedures in the ETH Zürich laboratory were performed by standard procedures[29].

S.7. Description of age, plant composition and decomposition degree variation in peat profiles

Peatlands are defined as environments where more carbon accumulates as peat than is released during the slow decomposition of plant material, due to wet and partly anaerobic conditions[30]. Peatlands started forming in Eurasia after the ice age, approximately 12000 years ago and may have accumulated several meters of peat during their subsequent development[31,32]. The top layer of peat consists of living biomass and litter, and with increasing depth of a peat profile, the material becomes gradually more decomposed, and older in age (Figs. S6, S7). The plant composition present in a soil profile at different depths may vary due to natural succession occurring over decades, centuries and millennia at the sampling locations, as seen in soil profile examples from the Russian Arctic permafrost peatland Rogovaya where our sample APR was collected (Fig. S7). The peat accumulation rate depends on biogeophysical factors, such as, vegetation composition, climate, water table and decomposition rate. Generally, shallower peats are found in the Arctic compared to boreal environments[30]. In the Arctic, old organic material may occur close to the surface due to the low peat accumulation rate. For instance, peat dating to approximately 1340 to 5870 years at 11 to 32 cm depth was

found in five profiles at the Svalbard tundra site where our sample APS was collected[33], and over 2000 years old peat at up to 39 cm depth at the Russian permafrost peatland from which our sample APR was collected[34], as shown in Figs. S6 and S7. The combustion experiments were performed on peat profiles that were not investigated for plant macrofossils or dated per depth from both sites, but the available data (Figs. S6 and S7) from other profiles collected at the respective sites[33,34] is indicative of the characteristics of our combustion experiment samples APS and APR. For instance, as at Rogovaya many peat profiles reached an age of approximately 2000 years (BP) at a depth of 20 to 40 cm, an average age of 2400 years for the APR profile covering the depth of 0-50 cm (Table S4), is reasonable.

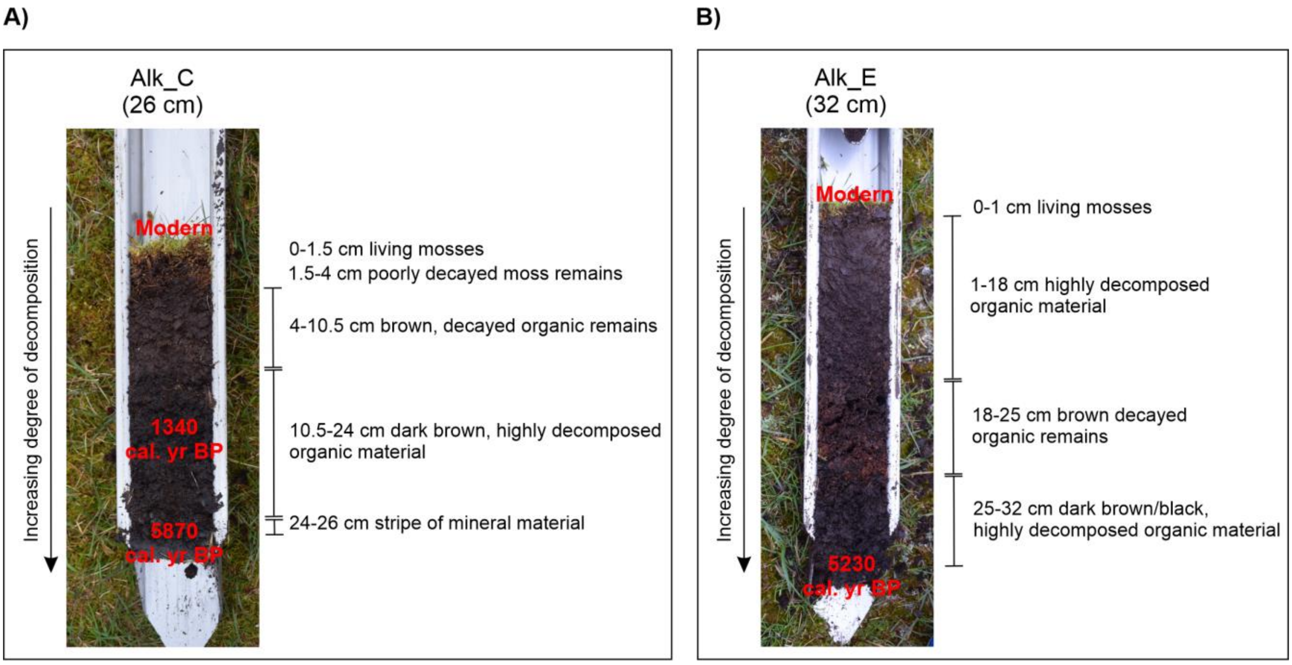

Figure S6. Two peat profiles collected at Alkhornet tundra, Svalbard (78°12'48.7"N 13°49'36.1"E), and their field description. The increasing degree of decomposition with depth is visible in both cores. Radiocarbon dating is available for the 15 cm depth in core Alk_C (A) and for the basal (bottom before hitting mineral soil or permafrost) peat for both profiles Alk_C and Alk_E (B) as calibrated years before present (BP, year 1950)[33].

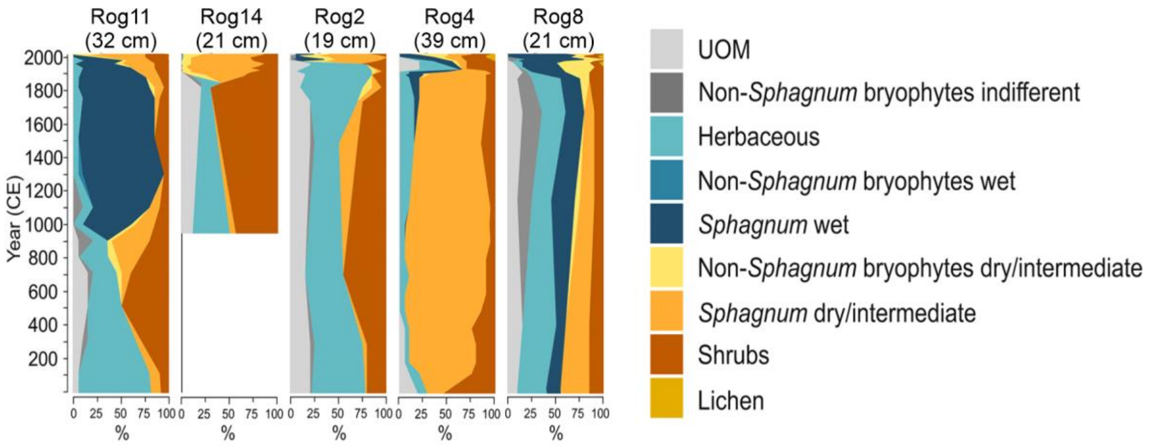

Figure S7. Vegetation composition changes over the last 2000 years in peat profiles collected at a permafrost peatland in Rogovaya, Komi Republic, Russia (67°19'43.0"N 62°36'18.1"E). Five profiles of different total depths are presented. The plant composition was determined by palaeoecological methodology as presented in Piilo et al.[34], and the dating is given as calibrated calendar years CE (Common Era). Modified from Piilo et al.[34]

Table S4. Radiocarbon ($\Delta^{14}C$) fraction modern and uncertainty as percentages (+/- %), and the age of original biomass samples and produced PM (Total Carbon and EC fraction) on filters from the combustion experiments.

	**Biomass**			**Particulate matter filter**					
				**Total Carbon (TC)**			**EC fraction**		
**Sample name**	**$F^{14}C$**	**(+/- %)**	**Age (y)**	**$F^{14}C$**	**(+/- %)**	**Age (y)**	**$F^{14}C$**	**(+/- %)**	**Age (y)**
1. *Sphagnum* peat (SPH)	1.01	0.82	> modern	0.785	11.7	1983 +/-1000 (n=2)	0.96	10.23	302 +/- 867
2. *Carex* peat (CRX)	1.06	0.88	> modern	1.10	1.47	> modern (n=2)	1.03	4.28	> modern
3. Shrub peat (SHR)	*na*	*na*	*na*	1.04	2.24	> modern (n=3)	1.02	1.88	> modern
4a. Commercial peat (CPFa)	0.83	0.85	1481 +/- 69	0.56	5.21	4618 +/- 430	0.55	2.95	4785 +/- 240
4b. Commercial peat (CPFb)	0.83	0.85	1481 +/- 69	0.58	3.84	4372 +/- 314	0.55	2.02	4738 +/- 163
4c. Commercial peat (CPS)	0.83	0.85	1481 +/- 69	0.57	1.42	4571 +/-115	0.58	5.45	4329 +/- 450
5. Finnish boreal peat (Lakkasuo) (FBL)	1.21	0.75	> modern	0.81	1.39	1683 +/- 113	0.75	1.78	*2273 +/- 144
6. Finnish boreal peat (Siikaneva) (FBS)	1.17	0.75	> modern	*na*	*na*	*na*	*na*	*na*	*na*
7. Arctic permafrost peat (Svalbard) (APS)	0.84	0.79	1408 +/- 63	0.98	1.16	171 +/-93	*na*	*na*	*na*
8. Arctic permafrost peat (Russia) (APR)	0.74	0.85	2435 +/- 69	0.81	1.22	1682 +/- 99	0.81	8.17	1663 +/- 684
9. Boreal forest surface (BFS)	*na*	*na*	*na*	1.09	1.14	> modern	1.09	2.84	> modern
10. Savanna (SAV)	0.99	0.79	48 +/- 63	*na*	*na*	*na*	0.97	0.85	259 +/- 68

* The radiocarbon analyses are made from a filter produced during a separate combustion round (of triplicate experiments) of the same original biomass as the reported values for the TC fraction.

Table S5. Stable carbon isotope ($\delta^{13}C$) values and uncertainty (standard deviation) for the original biomass and the particulate Total Cabon (TC) and Elemental Carbon (EC) fractions collected on filters during combustion

	**Biomass**		**Particulate matter filter**			
**Sample name**	**$\delta^{13}C$ (‰)**	**SD (‰)**	**TC $\delta^{13}C$ (‰)**	**SD ‰**	**EC $\delta^{13}C$ (‰)**	**SD ‰**
1. *Sphagnum* peat (SPH)	-30.13	0.2	*na*	*na*	*na*	*na*
2. *Carex* peat (CRX)	-29.66	0.15	*na*	*na*	-28.67	0.21
3. Shrub peat (SHR)	*na*	*na*	-28.94	0.12	-29.84	0.13
4a. Commercial peat (CPFa)	-27.89	0.2	-28.81	0.3	-27.6	0.2
4b. Commercial peat (CPFb)	-27.89	0.2	-30.45	0.2	-28.22	0.14
4c. Commercial peat (CPS)	-27.89	0.2	-30.03	0.12	*na*	*na*
5. Finnish boreal peat (Lakkasuo) (FBL)	-27.23	0.1	-25.29	0.1	-26.3 *	0.1
6. Finnish boreal peat (Siikaneva) (FBS)	-25.23	0.13	-26.77	0.12	-25.8	0.12
7. Arctic permafrost peat (Svalbard) (APS)	-26.15	0.12	-29.81	0.15	*na*	*na*
8. Arctic permafrost peat (Russia) (APR)	-28.58	0.11	-29.12	0.13	-26.67	0.13
9. Boreal forest surface (BFS)	*na*	*na*	-28.55	0.11	-28.44	0.2
10. Savanna (SAV)	-25.62	0.13	-28.23	0.12	-26.02	0.12

* The $\delta^{13}C$ analyses are made from a filter produced during a separate combustion round (of triplicate experiments) of the same original biomass than the reported values for the TC fraction.

References:

(1) Schneider, E.; Rüger, C. P.; Chacón-Patiño, M. L.; Somero, M.; Ruppel, M. M.; Ihalainen, M.; Köster, K.; Sippula, O.; Czech, H.; Zimmermann, R. The Complex Composition of Organic Aerosols Emitted during Burning Varies between Arctic and Boreal Peat. *Commun. Earth Environ.* **2024**, *5* (1), 137. https://doi.org/10.1038/s43247-024-01304-y.

(2) Mukherjee, A.; Hartikainen, A.; Somero, M.; Luostari, V.; Ihalainen, M.; Rüger, C. P.; Kekäläinen, T.; Nissinen, V. H.; Barreira, L. M. F.; Koponen, H.; Kokkola, T.; Li, D.; Vettikkat, L.; Yli-Pirilä, P.; Shahzaib, M.; Ruppel, M. M.; Vakkari, V.; Jaars, K.; Siebert, S. J.; Buchholz, A.; Köster, K.; Van Zyl, P. G.; Timonen, H.; Kinnunen, N.; Jänis, J.; Virtanen, A.; Virkkula, A.; Sippula, O. Brown Carbon Emissions from Laboratory Combustion of Eurasian Arctic-Boreal and South African Savanna Biomass. *Atmospheric Chem. Phys.* **2025**, *25* (22), 16747–16774. https://doi.org/10.5194/acp-25-16747-2025.

(3) Iinuma, Y.; Brüggemann, E.; Gnauk, T.; Müller, K.; Andreae, M. O.; Helas, G.; Parmar, R.; Herrmann, H. Source Characterization of Biomass Burning Particles: The Combustion of Selected European Conifers, African Hardwood, Savanna Grass, and German and Indonesian Peat. *J. Geophys. Res. Atmospheres* **2007**, *112* (D8), 2006JD007120. https://doi.org/10.1029/2006JD007120.

(4) Chakrabarty, R. K.; Gyawali, M.; Yatavelli, R. L. N.; Pandey, A.; Watts, A. C.; Knue, J.; Chen, L.-W. A.; Pattison, R. R.; Tsibart, A.; Samburova, V.; Moosmüller, H. Brown Carbon Aerosols from Burning of Boreal Peatlands: Microphysical Properties, Emission Factors, and Implications for Direct Radiative Forcing. *Atmospheric Chem. Phys.* **2016**, *16* (5), 3033–3040. https://doi.org/10.5194/acp-16-3033-2016.

(5) Rein, G. Smouldering Fires and Natural Fuels. In *Fire Phenomena and the Earth System*; Belcher, C. M., Ed.; Wiley, 2013; pp 15–33. https://doi.org/10.1002/9781118529539.ch2.

(6) Walker, X. J.; Rogers, B. M.; Veraverbeke, S.; Johnstone, J. F.; Baltzer, J. L.; Barrett, K.; Bourgeau-Chavez, L.; Day, N. J.; De Groot, W. J.; Dieleman, C. M.; Goetz, S.; Hoy, E.; Jenkins, L. K.; Kane, E. S.; Parisien, M.-A.; Potter, S.; Schuur, E. A. G.; Turetsky, M.; Whitman, E.; Mack, M. C. Fuel Availability Not Fire Weather Controls Boreal Wildfire Severity and Carbon Emissions. *Nat. Clim. Change* **2020**, *10* (12), 1130–1136. https://doi.org/10.1038/s41558-020-00920-8.

(7) Liu, C.; Chung, C. E.; Yin, Y.; Schnaiter, M. The Absorption Ångström Exponent of Black Carbon: From Numerical Aspects. *Atmospheric Chem. Phys.* **2018**, *18* (9), 6259–6273. https://doi.org/10.5194/acp-18-6259-2018.

(8) Rogers, B. M.; Soja, A. J.; Goulden, M. L.; Randerson, J. T. Influence of Tree Species on Continental Differences in Boreal Fires and Climate Feedbacks. *Nat. Geosci.* **2015**, *8* (3), 228–234. https://doi.org/10.1038/ngeo2352.

(9) Hu, Y.; Fernandez-Anez, N.; Smith, T. E. L.; Rein, G. Review of Emissions from Smouldering Peat Fires and Their Contribution to Regional Haze Episodes. *Int. J. Wildland Fire* **2018**, *27* (5), 293. https://doi.org/10.1071/WF17084.

(10) Feurdean, A. Experimental Production of Charcoal Morphologies to Discriminate Fuel Source and Fire Type: An Example from Siberian Taiga. *Biogeosciences* **2021**, *18* (12), 3805–3821. https://doi.org/10.5194/bg-18-3805-2021.

(11) Tissari, J.; Väätäinen, S.; Leskinen, J.; Savolahti, M.; Lamberg, H.; Kortelainen, M.; Karvosenoja, N.; Sippula, O. Fine Particle Emissions from Sauna Stoves: Effects of Combustion Appliance and Fuel, and Implications for the Finnish Emission Inventory. *Atmosphere* **2019**, *10* (12), 775. https://doi.org/10.3390/atmos10120775.

(12) Akagi, S. K.; Yokelson, R. J.; Wiedinmyer, C.; Alvarado, M. J.; Reid, J. S.; Karl, T.; Crounse, J. D.; Wennberg, P. O. Emission Factors for Open and Domestic Biomass Burning for Use in

Atmospheric Models. *Atmospheric Chem. Phys.* **2011**, *11* (9), 4039–4072. https://doi.org/10.5194/acp-11-4039-2011.
(13) Yokelson, R. J.; Goode, J. G.; Ward, D. E.; Susott, R. A.; Babbitt, R. E.; Wade, D. D.; Bertschi, I.; Griffith, D. W. T.; Hao, W. M. Emissions of Formaldehyde, Acetic Acid, Methanol, and Other Trace Gases from Biomass Fires in North Carolina Measured by Airborne Fourier Transform Infrared Spectroscopy. *J. Geophys. Res. Atmospheres* **1999**, *104* (D23), 30109–30125. https://doi.org/10.1029/1999JD900817.
(14) Birch, M. E.; Cary, R. A. Elemental Carbon-Based Method for Monitoring Occupational Exposures to Particulate Diesel Exhaust. *Aerosol Sci. Technol.* **1996**, *25* (3), 221–241. https://doi.org/10.1080/02786829608965393.
(15) Cavalli, F.; Viana, M.; Yttri, K. E.; Genberg, J.; Putaud, J.-P. Toward a Standardised Thermal-Optical Protocol for Measuring Atmospheric Organic and Elemental Carbon: The EUSAAR Protocol. *Atmospheric Meas. Tech.* **2010**, *3* (1), 79–89. https://doi.org/10.5194/amt-3-79-2010.
(16) Huang, L.; Zhang, W.; Santos, G. M.; Rodríguez, B. T.; Holden, S. R.; Vetro, V.; Czimczik, C. I. Application of the ECT9 Protocol for Radiocarbon-Based Source Apportionment of Carbonaceous Aerosols. *Atmospheric Meas. Tech.* **2021**, *14* (5), 3481–3500. https://doi.org/10.5194/amt-14-3481-2021.
(17) Chow, J. C.; Watson, J. G.; Chen, L.-W. A.; Chang, M. C. O.; Robinson, N. F.; Trimble, D.; Kohl, S. The IMPROVE_A Temperature Protocol for Thermal/Optical Carbon Analysis: Maintaining Consistency with a Long-Term Database. *J. Air Waste Manag. Assoc.* **2007**, *57* (9), 1014–1023. https://doi.org/10.3155/1047-3289.57.9.1014.
(18) Chen, L.-W. A.; Chow, J. C.; Wang, X. L.; Robles, J. A.; Sumlin, B. J.; Lowenthal, D. H.; Zimmermann, R.; Watson, J. G. Multi-Wavelength Optical Measurement to Enhance Thermal/Optical Analysis for Carbonaceous Aerosol. *Atmospheric Meas. Tech.* **2015**, *8* (1), 451–461. https://doi.org/10.5194/amt-8-451-2015.
(19) Chow, J. C.; Watson, J. G.; Green, M. C.; Wang, X.; Chen, L.-W. A.; Trimble, D. L.; Cropper, P. M.; Kohl, S. D.; Gronstal, S. B. Separation of Brown Carbon from Black Carbon for IMPROVE and Chemical Speciation Network $PM_{2.5}$ Samples. *J. Air Waste Manag. Assoc.* **2018**, *68* (5), 494–510. https://doi.org/10.1080/10962247.2018.1426653.
(20) Chow, J. C.; Chen, L.-W. A.; Wang, X.; Green, M. C.; Watson, J. G. Improved Estimation of PM2.5 Brown Carbon Contributions to Filter Light Attenuation. *Particuology* **2021**, *56*, 1–9. https://doi.org/10.1016/j.partic.2021.01.001.
(21) Chakrabarty, R. K.; Shetty, N. J.; Thind, A. S.; Beeler, P.; Sumlin, B. J.; Zhang, C.; Liu, P.; Idrobo, J. C.; Adachi, K.; Wagner, N. L.; Schwarz, J. P.; Ahern, A.; Sedlacek, A. J.; Lambe, A.; Daube, C.; Lyu, M.; Liu, C.; Herndon, S.; Onasch, T. B.; Mishra, R. Shortwave Absorption by Wildfire Smoke Dominated by Dark Brown Carbon. *Nat. Geosci.* **2023**, *16* (8), 683–688. https://doi.org/10.1038/s41561-023-01237-9.
(22) Lin, P.; Aiona, P. K.; Li, Y.; Shiraiwa, M.; Laskin, J.; Nizkorodov, S. A.; Laskin, A. Molecular Characterization of Brown Carbon in Biomass Burning Aerosol Particles. *Environ. Sci. Technol.* **2016**, *50* (21), 11815–11824. https://doi.org/10.1021/acs.est.6b03024.
(23) Saleh, R.; Cheng, Z.; Atwi, K. The Brown–Black Continuum of Light-Absorbing Combustion Aerosols. *Environ. Sci. Technol. Lett.* **2018**, *5* (8), 508–513. https://doi.org/10.1021/acs.estlett.8b00305.
(24) Adler, G.; Wagner, N. L.; Lamb, K. D.; Manfred, K. M.; Schwarz, J. P.; Franchin, A.; Middlebrook, A. M.; Washenfelder, R. A.; Womack, C. C.; Yokelson, R. J.; Murphy, D. M. Evidence in Biomass Burning Smoke for a Light-Absorbing Aerosol with Properties Intermediate between Brown and Black Carbon. *Aerosol Sci. Technol.* **2019**, *53* (9), 976–989. https://doi.org/10.1080/02786826.2019.1617832.
(25) Ciesielczuk, J.; Górka, M.; Fabiańska, M. J.; Misz-Kennan, M.; Jura, D. The Influence of Heating on the Carbon Isotope Composition, Organic Geochemistry and Petrology of Coal

from the Upper Silesian Coal Basin (Poland): An Experimental and Field Study. *Int. J. Coal Geol.* **2021**, *241*, 103749. https://doi.org/10.1016/j.coal.2021.103749.
(26) Widory, D. Combustibles, Fuels and Their Combustion Products: A View through Carbon Isotopes. *Combust. Theory Model.* **2006**, *10* (5), 831–841. https://doi.org/10.1080/13647830600720264.
(27) Vernooij, R.; Dusek, U.; Popa, M. E.; Yao, P.; Shaikat, A.; Qiu, C.; Winiger, P.; Van Der Veen, C.; Eames, T. C.; Ribeiro, N.; Van Der Werf, G. R. Stable Carbon Isotopic Composition of Biomass Burning Emissions – Implications for Estimating the Contribution of C_3 and C_4 Plants. *Atmospheric Chem. Phys.* **2022**, *22* (4), 2871–2890. https://doi.org/10.5194/acp-22-2871-2022.
(28) Huang, L.; Brook, J. R.; Zhang, W.; Li, S. M.; Graham, L.; Ernst, D.; Chivulescu, A.; Lu, G. Stable Isotope Measurements of Carbon Fractions (OC/EC) in Airborne Particulate: A New Dimension for Source Characterization and Apportionment. *Atmos. Environ.* **2006**, *40* (15), 2690–2705. https://doi.org/10.1016/j.atmosenv.2005.11.062.
(29) McIntyre, C. P.; Wacker, L.; Haghipour, N.; Blattmann, T. M.; Fahrni, S.; Usman, M.; Eglinton, T. I.; Synal, H.-A. Online^{13} C and^{14} C Gas Measurements by EA-IRMS–AMS at ETH Zürich. *Radiocarbon* **2017**, *59* (3), 893–903. https://doi.org/10.1017/RDC.2016.68.
(30) Rydin, H.; Jeglum, J. K.; Bennett, K. D. *The Biology of Peatlands*, 2nd ed.; Biology of habitats; Oxford University Press: Oxford, 2013.
(31) Loisel, J.; Yu, Z.; Beilman, D. W.; Camill, P.; Alm, J.; Amesbury, M. J.; Anderson, D.; Andersson, S.; Bochicchio, C.; Barber, K.; Belyea, L. R.; Bunbury, J.; Chambers, F. M.; Charman, D. J.; De Vleeschouwer, F.; Fiałkiewicz-Kozieł, B.; Finkelstein, S. A.; Gałka, M.; Garneau, M.; Hammarlund, D.; Hinchcliffe, W.; Holmquist, J.; Hughes, P.; Jones, M. C.; Klein, E. S.; Kokfelt, U.; Korhola, A.; Kuhry, P.; Lamarre, A.; Lamentowicz, M.; Large, D.; Lavoie, M.; MacDonald, G.; Magnan, G.; Mäkilä, M.; Mallon, G.; Mathijssen, P.; Mauquoy, D.; McCarroll, J.; Moore, T. R.; Nichols, J.; O'Reilly, B.; Oksanen, P.; Packalen, M.; Peteet, D.; Richard, P. J.; Robinson, S.; Ronkainen, T.; Rundgren, M.; Sannel, A. B. K.; Tarnocai, C.; Thom, T.; Tuittila, E.-S.; Turetsky, M.; Väliranta, M.; van der Linden, M.; van Geel, B.; van Bellen, S.; Vitt, D.; Zhao, Y.; Zhou, W. A Database and Synthesis of Northern Peatland Soil Properties and Holocene Carbon and Nitrogen Accumulation. *The Holocene* **2014**, *24* (9), 1028–1042. https://doi.org/10.1177/0959683614538073.
(32) Ruppel, M.; Väliranta, M.; Virtanen, T.; Korhola, A. Postglacial Spatiotemporal Peatland Initiation and Lateral Expansion Dynamics in North America and Northern Europe. *The Holocene* **2013**, *23* (11), 1596–1606. https://doi.org/10.1177/0959683613499053.
(33) Juselius, T.; Ravolainen, V.; Zhang, H.; Piilo, S.; Müller, M.; Gallego-Sala, A.; Väliranta, M. Newly Initiated Carbon Stock, Organic Soil Accumulation Patterns and Main Driving Factors in the High Arctic Svalbard, Norway. *Sci. Rep.* **2022**, *12* (1), 4679. https://doi.org/10.1038/s41598-022-08652-9.
(34) Piilo, S. R.; Väliranta, M. M.; Amesbury, M. J.; Aquino-López, M. A.; Charman, D. J.; Gallego-Sala, A.; Garneau, M.; Koroleva, N.; Kärppä, M.; Laine, A. M.; Sannel, A. B. K.; Tuittila, E.; Zhang, H. Consistent Centennial-scale Change in European SUB-ARCTIC Peatland Vegetation toward *Sphagnum* Dominance—Implications for Carbon Sink Capacity. *Glob. Change Biol.* **2023**, *29* (6), 1530–1544. https://doi.org/10.1111/gcb.16554.
